# SARS-CoV-2 impairs interferon production via NSP2-induced repression of mRNA translation

**DOI:** 10.1073/pnas.2204539119

**Published:** 2022-07-25

**Authors:** Zhang Xu, Jung-Hyun Choi, David L. Dai, Jun Luo, Reese Jalal Ladak, Qian Li, Yimeng Wang, Christine Zhang, Shane Wiebe, Alex C. H. Liu, Xiaozhuo Ran, Jiaqi Yang, Parisa Naeli, Aitor Garzia, Lele Zhou, Niaz Mahmood, Qiyun Deng, Mohamed Elaish, Rongtuan Lin, Lara K. Mahal, Tom C. Hobman, Jerry Pelletier, Tommy Alain, Silvia M. Vidal, Thomas Duchaine, Mohammad T. Mazhab-Jafari, Xiaojuan Mao, Seyed Mehdi Jafarnejad, Nahum Sonenberg

**Affiliations:** ^a^Rosalind and Morris Goodman Cancer Institute, McGill University, Montreal, QC, H3A 1A3, Canada;; ^b^Department of Biochemistry, McGill University, Montreal, QC, H3A 1A3, Canada;; ^c^Princess Margaret Cancer Centre, University Health Network, University of Toronto, Toronto, ON M5G 2C1, Canada;; ^d^Meakins-Christie Laboratories and Respiratory Division, McGill University, Montreal, QC, H4A 3J1, Canada;; ^e^Lady Davis Institute, Department of Medicine, McGill University, Montreal, QC, H3T 1E2, Canada;; ^f^Department of Medical Microbiology, Faculty of Medicine, University of Manitoba, Winnipeg, MB, R3T 2N2, Canada;; ^g^Patrick G Johnston Centre for Cancer Research, Queen’s University Belfast, Belfast, Northern Ireland, BT9 7AE, United Kingdom;; ^h^Laboratory for RNA Molecular Biology, The Rockefeller University, New York, NY 10065;; ^i^Department of Chemistry, University of Alberta, Edmonton, AB T6G 2G2, Canada;; ^j^Department of Cell Biology, University of Alberta, Edmonton, AB T6G 2H7, Canada;; ^k^Poultry Diseases Department, Faculty of Veterinary Medicine, Cairo University, Giza, 12613 Egypt;; ^l^Children’s Hospital of Eastern Ontario Research Institute, Department of Biochemistry, Microbiology and Immunology, University of Ottawa, Ottawa, ON, K1H 5B2, Canada;; ^m^Department of Human Genetics, McGill University, Montreal, QC, H3G 0B1 Canada

**Keywords:** SARS-CoV-2, mRNA translation, 4EHP, NSP2, GIGYF2

## Abstract

Viruses evade the innate immune response by suppressing the production or activity of cytokines such as type I interferons (IFNs). Here we report the discovery of a mechanism by which the SARS-CoV-2 virus coopts an intrinsic cellular machinery to suppress the production of the key immunostimulatory cytokine IFN-β. We reveal that the SARS-CoV-2 encoded nonstructural protein 2 (NSP2) directly interacts with the cellular GIGYF2 protein. This interaction enhances the binding of GIGYF2 to the mRNA cap-binding protein 4EHP, thereby repressing the translation of the *Ifnb1* mRNA. Depletion of GIGYF2 or 4EHP significantly enhances IFN-β production, which inhibits SARS-CoV-2 replication. Our findings reveal a target for rescuing the antiviral innate immune response to SARS-CoV-2 and other RNA viruses.

## Significance

A robust antiviral innate immune response is indispensable for combating infections. However, an exacerbated response can result in pathological inflammation and tissue damage. mRNA translational control mechanisms play a crucial role in maintaining the appropriate magnitude and duration of the immune response. We show that the GIGYF2/4EHP translational repressor complex represses translation of *Ifnb1* mRNA, which encodes type I interferon β (IFN-β). We also demonstrate that the NSP2 protein encoded by SARS-CoV-2 virus further impedes translation of *Ifnb1* mRNA through coopting the GIGYF2/4EHP complex, leading to evasion of a cellular innate immune response. The knowledge of the mechanism of action of NSP2-mediated IFN-β suppression provides valuable information for development of treatments for infections of SARS-CoV-2 and other coronaviruses.

Production of type I interferons (IFN-α and IFN-β) is pivotal to antiviral immunity as a host defense mechanism (1). Replication of SARS-CoV-2 is sensitive to type I IFN in vitro (2–4), and SARS-CoV-2 infection in humans is associated with a deficiency in type I IFN response (5, 6). In the early phase of the SARS-CoV-2 infection, a robust IFN-induced antiviral response limits viral replication and prevents severe COVID-19 illness (7, 8). Conversely, impaired production of type I IFN is associated with higher viral titers in blood and pernicious symptoms in late-stage SARS-CoV-2–infected patients (9).

Production of type I IFNs is controlled at several levels, including transcription and translation. Notably, multiple SARS-CoV-2 proteins inhibit *Ifnb1* transcription, including NSP1, 3, 5, 6, 12, 13, 14, 15, ORF3a, ORF6, and ORF7b (10, 11). Potent translational repression of *Ifnb1* mRNA is also manifested during SARS-CoV-2 infection (12). Although SARS-CoV-2 represses general cellular mRNA translation machinery to support viral mRNA translation (12–14), specific repression of *Ifnb1* mRNA translation was not reported.

Translation of most eukaryotic mRNAs is facilitated by binding of the eukaryotic initiation factor 4E (eIF4E) to the 5′ cap structure (m7GpppN, where N is any nucleotide, and m is a methyl group). eIF4E binds to the cap structure as a subunit of the eIF4F complex, which also contains the scaffolding protein eIF4G and the RNA helicase eIF4A (15). Being the least abundant initiation factor, eIF4E is rate limiting for eIF4F formation and translation initiation. The eIF4E homologous protein, 4EHP (eIF4E2) binds the cap structure but fails to initiate canonical translation because it does not interact with eIF4G. Consequently, 4EHP represses translation upon recruitment to target mRNAs (e.g., via 4E-T protein upon recruitment by microRNAs) (16–19). GIGYF2 (Grb10-interacting GYF [glycine, tyrosine, phenylalanine] protein 2) is another protein that directly interacts with 4EHP to inhibit mRNA translation or decrease mRNA stability (19–24). GIGYF2 participates in both 4EHP-dependent and -independent posttranscriptional repression mechanisms (20, 22–24). We recently reported the 4EHP-mediated, miR-34a–directed translational repression of *Ifnb1* mRNA (25). This mechanism limits IFN-β production upon vesicular stomatitis virus infection, likely to prohibit prolonged inflammatory responses (25). Whether GIGYF2 is involved in the 4EHP-mediated translational repression of IFN-β production is unknown.

Several large-scale proteomic studies reported the interaction of SARS-CoV-2 nonstructural protein 2 (NSP2) with 4EHP and GIGYF2 (26–28). Here, we document a mechanism by which the NSP2 protein impedes IFN-β expression through translational repression of *Ifnb1* mRNA by coopting the GIGYF2/4EHP complex, leading to evasion of a cellular innate immune response and enhanced viral replication.

## Results

### NSP2 Specifically Interacts with GIGYF2 in the GIGYF2/4EHP Translation Repression Complex.

**Fig. 1.**
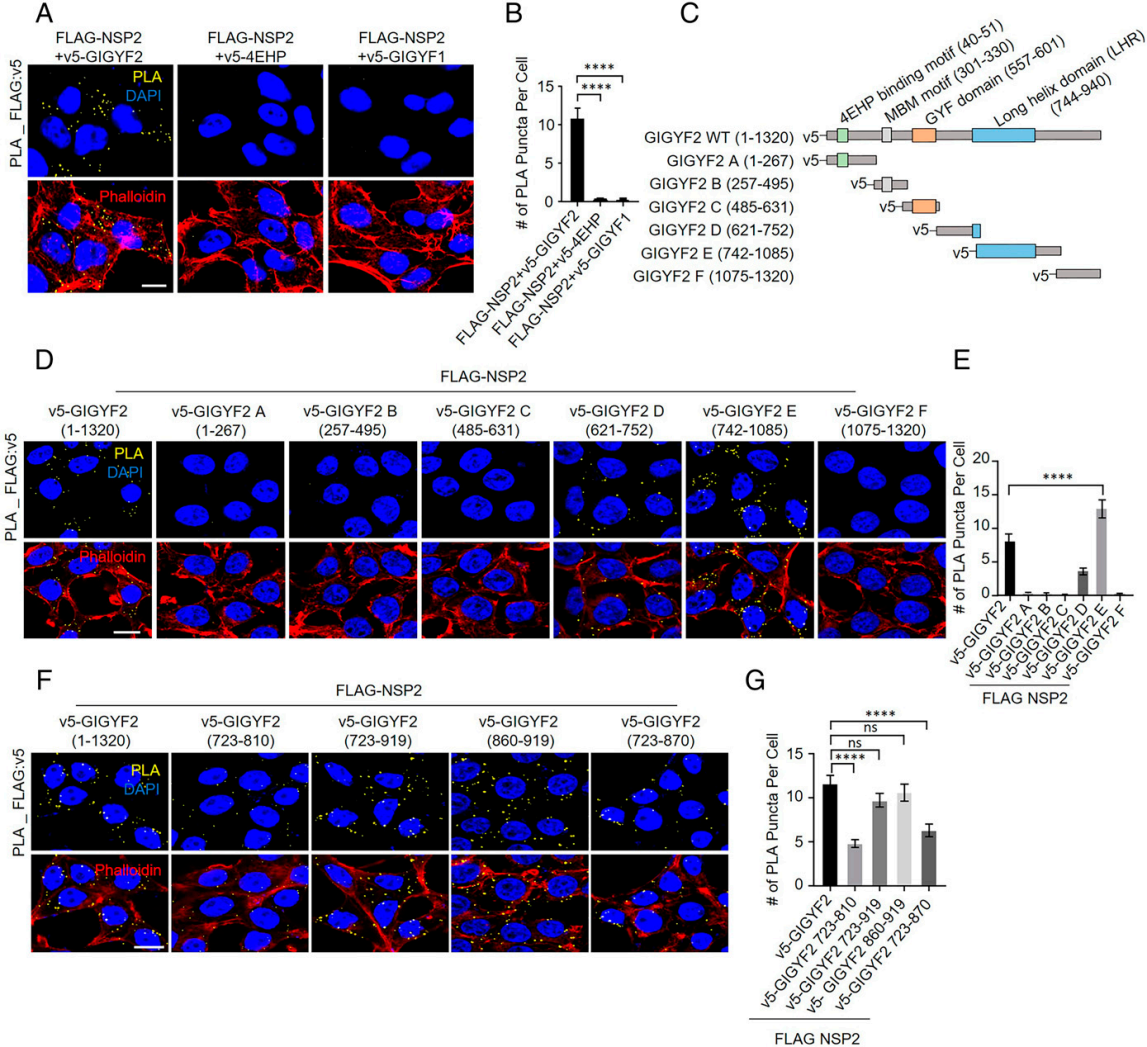
Characterization of the NSP2 interaction with the GIGYF2/4EHP complex. (*A*) PLA for detection of NSP2–GIGYF2. Sites of interactions are visible as fluorescent punctate in HEK293T cells transfected with vectors expressing v5-tagged GIGYF2, 4EHP, or GIGYF1 together with FLAG-NSP2. At 24 h posttransfection, cells were fixed and subjected to PLA using FLAG and v5 antibodies. PLA signals are shown in yellow. Nucleus and actin cytoskeleton were counterstained with DAPI (blue) and phalloidin (red), respectively. (Scale bar, 10 µm.) (*B*) Quantification of positive PLA signals in *A*. The number of PLA signals from at least 30 cells was counted in each sample. Data are presented as mean ± SD (*n* = 5). (*C*) Schematic of the primary structures of FL GIGYF2 and fragments A through F used in this study. (*D*) HEK293T cells were cotransfected with FLAG-NSP2 and GIGYF2 fragment constructs shown in *E* or full-length GIGYF2 (as control). The 24-h posttransfection cells were fixed and subjected to PLA using FLAG and v5 antibodies. PLA signals are shown in yellow. Nucleus and actin cytoskeleton were counterstained with DAPI (blue) and phalloidin (red), respectively. (Scale bar, 10 µm.) (*E*) Quantification of positive PLA signals in *D*. The number of PLA signals from at least 20 cells was counted in each sample. *n* = 3 independent experiments. (*F*) PLA assay for testing the interactions between NSP2 and the indicated fragments of GIGYF2-LHR, as described in *D*. (*G*) Quantification of positive PLA signals from *F*. The number of PLA signals from at least 20 cells was counted in each sample. Data are presented as mean ± SD (*n* = 5). ns, nonsignificant, ****P < 0.0001, one-way analysis of variance (ANOVA) with Bonferroni’s post hoc test. See also *SI Appendix*, Fig. S1.

We first sought to confirm the interaction between NSP2 and the GIGYF2/4EHP complex, which was reported in high-throughput surveys (26–28). We visualized the interaction of NSP2 with the GIGYF2/4EHP complex in cells using proximity ligation assay (PLA). Cotransfection of FLAG-NSP2 with v5-GIGYF2 resulted in a strong PLA signal, which was absent in cells cotransfected with FLAG-NSP2 and v5-GIGYF1 (a paralogue of GIGYF2) (Fig. 1 *A* and *B* and *SI Appendix*, Fig. S1 *A* and *B*). Strikingly, we did not detect any signal upon cotransfection of FLAG-NSP2 with v5-4EHP (Fig. 1 *A* and *B*), which indicates that NSP2 interacts directly with GIGYF2, but not with 4EHP. Coimmunoprecipitation (co-IP) experiments showed that GIGYF2 and 4EHP coprecipitated with FLAG-tagged NSP2 (FLAG-NSP2), whereas no GIGYF2 or 4EHP pulldown was detected with FLAG-GFP or FLAG-NSP1 baits (*SI Appendix*, Fig. S1*C*). Notably, while the lower level of expression of NSP1 compared with NSP2 may explain the lack of coprecipitation of 4EHP/GIGYF2 with NSP1 in our co-IP assay, the interaction between NSP2 and 4EHP/GIGYF2 has been consistently observed in several high-throughput analyses by other groups (26–28), but interaction with 4EHP/GIGYF2 has never been reported for NSP1 or other SARS-CoV-2–encoded proteins.

We next mapped the region of GIGYF2 responsible for binding NSP2. The human GIGYF2 cDNA portions were expressed as six v5-tagged contiguous fragments (GIGYF2 A–F; Fig. 1*C*), and cotransfected with FLAG-tagged NSP2 in HEK293T cells. We observed an interaction of NSP2 with full-length (FL) GIGYF2 and fragment E (742–1,085) in PLA assays (Fig. 1 *D* and *E* and *SI Appendix*, Fig. S1*D*), and to a lesser extent with the partially overlapping GIGYF2-D fragment (621–752). In agreement with the PLA results, IP with the anti-FLAG followed by blotting with anti-v5 antibody revealed that GIGYF2-FL (1–1,320), GIGYF2-E (742–1,085), and to a far lesser extent GIGYF2-A (1–267), interacted with FLAG-NSP2 (*SI Appendix*, Fig. S1*E*). Notably, our PLA and IP assays showed a slight but significant increase in binding of NSP2 to fragment E compared to the full-length GIGYF2, which may be due to a small increase in expression of fragment E compared with the full-length GIGYF2 as evident in parallel Western blots (*SI Appendix*, Fig. S1 *D* and *E*). These data demonstrate that the NSP2-interacting region of GIGYF2 spans residues 742–1,085. We further narrowed down the interaction site and revealed that the region spanning amino acids 860–919 of GIGYF2 interacts with NSP2 (Fig. 1 *F* and *G* and *SI Appendix*, Fig. S1*F*). This fragment is contained within a singular, long alpha helix region (LHR) (723–919), which is predicted by AlphaFold 2 (16, 29).

### NSP2 Induces Translational Repression by Bolstering GIGYF2–4EHP Interaction.

**Fig. 2.**
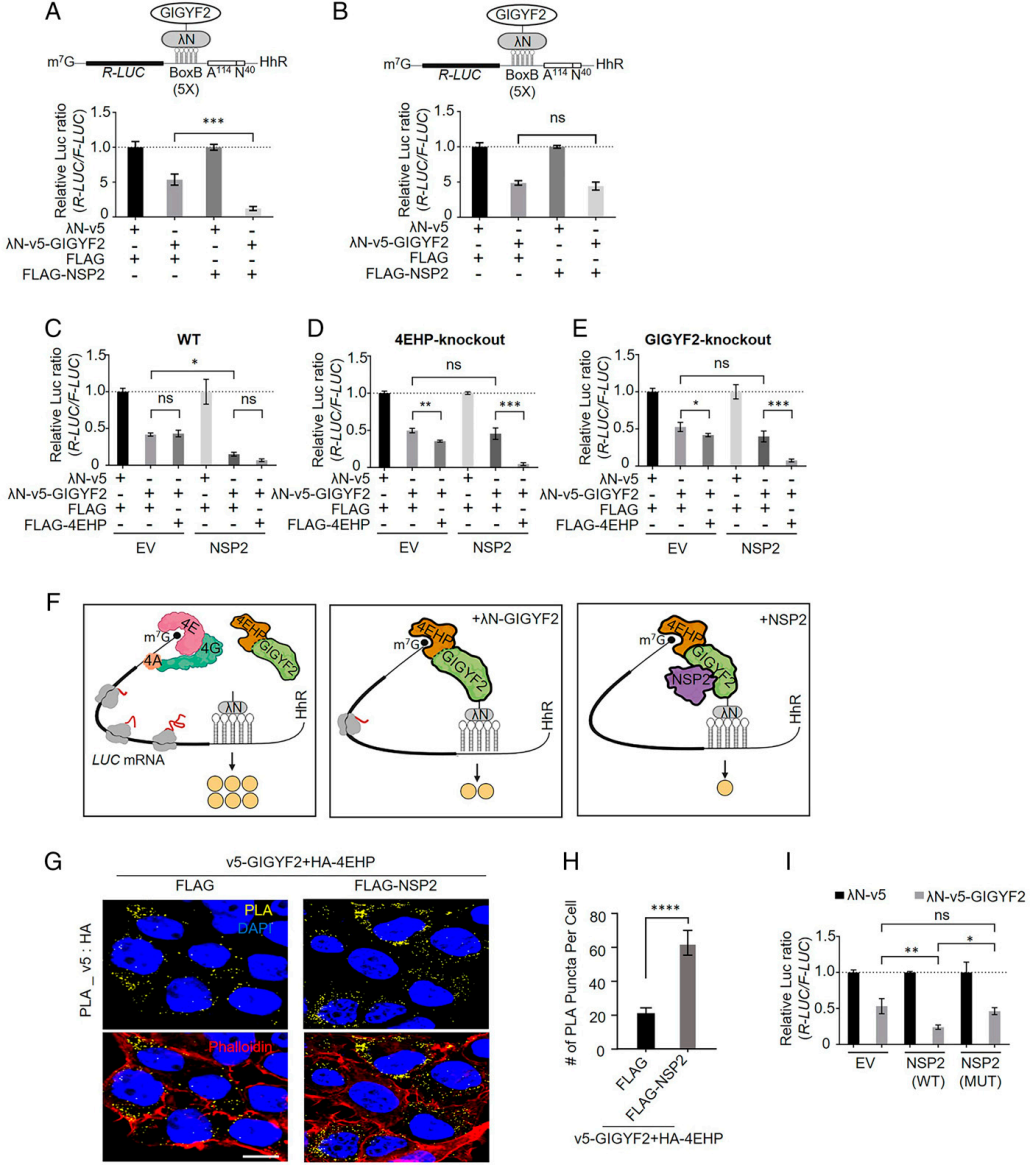
NSP2 augments GIGYF2/4EHP complex–mediated translational repression by enhancing the GIGYF2 interaction with 4EHP. (*A*) WT HEK293 cells were cotransfected with plasmids expressing either λN-v5-GIGYF2 or λN-v5 as control, along with *R-Luc*-5BoxB-A114-N40-HhR (hammerhead ribozyme) and *F-Luc* (as control), followed by dual-luciferase measurement assay. Data are presented as mean ± SD (*n* = 3). The schematic shows a graphic model of the λN-v5-GIGYF2 tethering system. (*B*) Analysis of λN-v5-GIGYF2 tethering-induced silencing in GIGYF2-KO cells that overexpress FLAG-NSP2. Data are presented as mean ± SD (*n* = 3). (*C*) WT cells were cotransfected with vectors expressing either λN-v5-GIGYF2 or λN-v5 control, along with *R-Luc*-5BoxB-A114-N40-HhR and *F-Luc* (as control), in combination with FLAG-4EHP or FLAG-empty plasmids. A dual-luciferase assay was performed 24 h posttransection. Data are presented as mean ± SD (*n* = 3). (*D*–*E*) GIGYF2-tethering assay carried out in 4EHP-KO cells in *D* and GIGYF2-KO cells in *E*. Data are presented as mean ± SD (*n* = 3). (*F*) Graphic illustration of the GIGYF2/4EHP-mediated induction of translational repression by NSP2. (*G*) PLA assay for detection of GIGYF2–4EHP interactions in HEK293T cells transfected with vectors expressing v5-GIGYF2 and HA-4EHP together with FLAG-empty or FLAG-NSP2. Cells were fixed and subjected to PLA using v5 and HA antibodies 24 h posttransfection. (Scale bar, 10 µm.) (*H*) Quantification of positive PLA signals from *G*. (*I*) WT cells were cotransfected with either λN-v5-GIGYF2 or λN-v5 control vector along with *R-Luc*-5BoxB-A114-N40-HhR and *F-Luc* (as control), in combination with FLAG-EV, wild-type FLAG-NSP2, or FLAG-NSP2^G262V/G265V^-expressing plasmids. A dual-luciferase assay was performed 24 h posttransection. Data are presented as mean ± SD (*n* = 3). The number of PLA signals from at least 20 cells was counted in each sample. *n* = 5 independent experiments. Data are presented as mean ± SD (*n* = 5). ns, nonsignificant, *P < 0.05, **P < 0.01, ***P < 0.001, ****P < 0.0001, one-way ANOVA with Bonferroni’s post hoc test. See also *SI Appendix*, Figs. S2 and S3.

GIGYF2 employs both 4EHP-dependent and -independent mechanisms to translationally repress target mRNAs (20, 23, 30). To investigate whether NSP2 impacts translational repression by GIGYF2, we used the λN-BoxB system to tether the λN-fused GIGYF2 to the 3′ untranslated region (3′ UTR) of *Renilla* luciferase (*R-Luc*) mRNA. The reporter mRNA is protected from deadenylation by a hammerhead ribozyme (HhR) located at its 3′ end (31, 32). We cotransfected the reporter along with FLAG-NSP2 or FLAG control plasmid. While GIGYF2 tethering alone resulted in ∼50% repression (the *R-Luc*/*F-Luc* [*firefly luciferase*] ratio) compared to the counterpart λN vector, coexpression of NSP2 along with GIGYF2-tethering plasmid resulted in a stronger repression (∼88%) (Fig. 2*A* and *SI Appendix*, Fig. S2*A*). In contrast, *R-Luc* repression was unaffected in GIGYF2-knockout (KO) cells transfected with NSP2 (Fig. 2*B* and *SI Appendix*, Fig. S2*B*), most probably due to the absence of 4EHP, which is rendered unstable in GIGYF2-depleted cells (20). Importantly, NSP2 did not increase translational repression by tethered GIGYF1 (*SI Appendix*, Fig. S2 *C* and *D*), demonstrating the specificity of NSP2-induced GIGYF2-mediated translational repression.

To study how 4EHP contributes to the GIGYF2-mediated translational repression by NSP2, GIGYF2 tethering experiments were carried out in wild-type (WT), 4EHP-KO, and GIGYF2-KO cells with or without ectopic 4EHP expression. Expression of NSP2 enhanced the GIGYF2 tethering–induced silencing (from *R-Luc*/*F-Luc* ratio 0.41 to 0.15) in WT cells, but not in 4EHP-KO or GIGYF2-KO cells (Fig. 2 *C–E* and *SI Appendix*, Fig. S2 *E*–*G*). 4EHP expression restored GIGYF2-mediated repression (from *R-Luc*/*F-Luc* ratio 0.45 to 0.05 and from 0.40 to 0.08 in NSP2-overexpressing 4EHP-KO and GIGYF2-KO cells, respectively). These results support the notion that NSP2 promotes GIGYF2-induced translational repression in a 4EHP-dependent manner (Fig. 2*F*). To determine whether NSP2 bolsters the interaction of GIGYF2 and 4EHP, we carried out the PLA assay following cotransfection of v5-GIGYF2 and HA-4EHP into WT HEK293 cells with NSP2. As expected, the interaction between 4EHP and GIGYF2 was dramatically enhanced (threefold) upon expression of NSP2 (FLAG: 19.8 ± 1.9, FLAG-NSP2: 61 ± 5.5 punctate per cell (Fig. 2 *G* and *H* and *SI Appendix*, Fig. S3 *A*–*C*).

Notably, natural variants of SARS-CoV-2 with glycine-to-valine point mutations at residues 262 and 265 in NSP2 have been reported (33). A recent study of changes in NSP2’s virus–host protein–protein interactome caused by naturally occurring mutations using affinity purification mass-spectrometry (AP-MS) assay found that the NSP2^G262V/G265V^ double mutant failed to interact with the GIGYF2 and 4EHP (28) . PLA and co-IP assays upon cotransfection of HEK293 cells with v5-GIGYF2 and either wild-type FLAG-NSP2 or the NSP2^G262V/G265V^ mutant confirmed that the G262V/G265V mutations impeded the NSP2 interaction with GIGYF2 (*SI Appendix*, Fig. S3 *D*–*G*). Importantly, GIGYF2 tethering experiments entailing cotransfecting the λN-v5 fused GIGYF2 with wild-type NSP2 or the NSP2^G262V/G265V^ mutants revealed that the G262V/G265V mutations significantly abrogated the NSP2-induced GIGYF2-mediated repression of the reporter mRNA (Fig. 2*I* and *SI Appendix*, Fig. S3*H*).

### The GIGYF2/4EHP Complex Represses IFN-β Production.

**Fig. 3.**
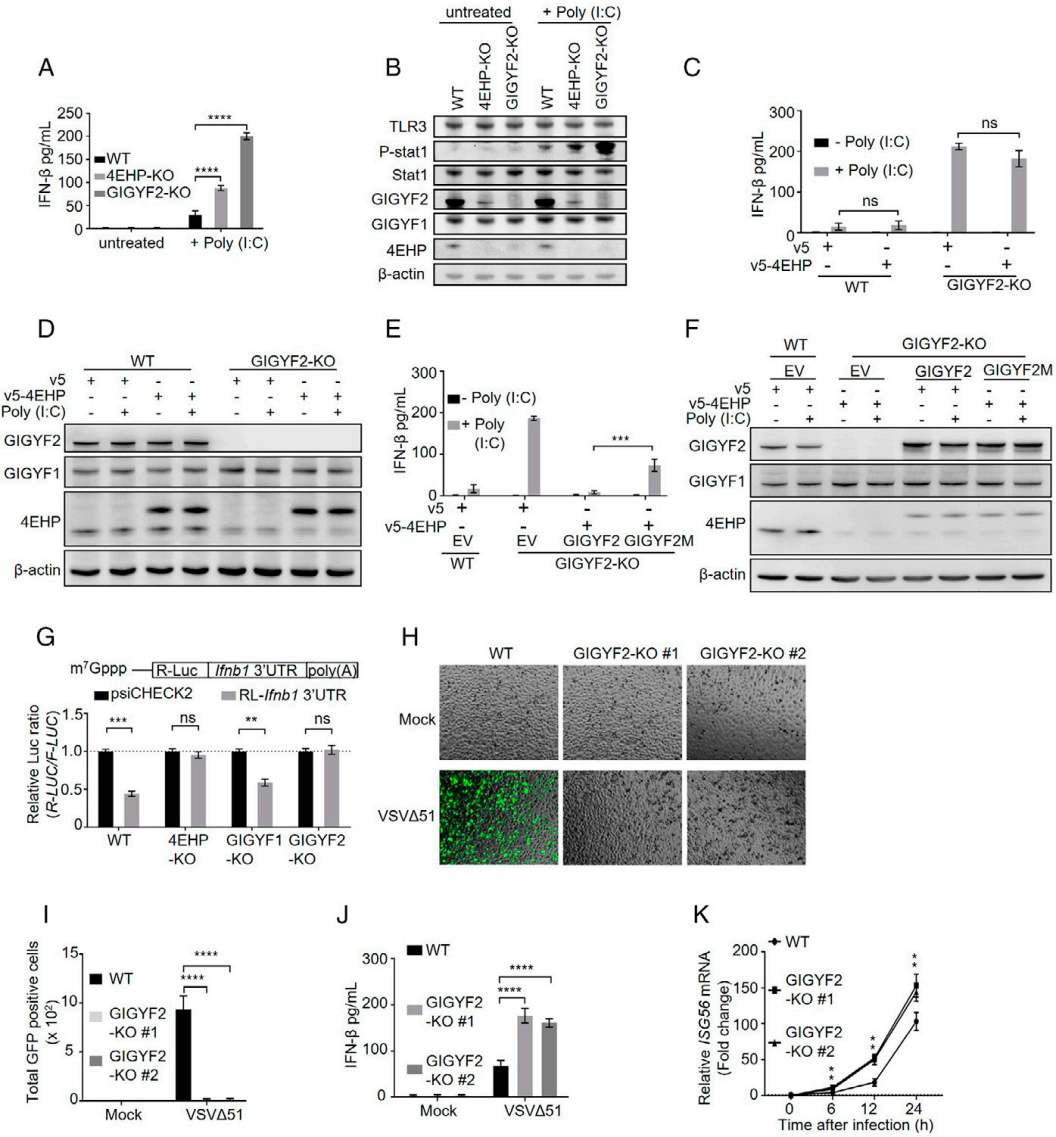
GIGYF2/4EHP complex formation is critical for repression of *Ifnb1* mRNA translation and enabling viral replication. (*A*) ELISA measurement of IFN-β production in WT, 4EHP-KO, and GIGYF2-KO HEK293 cells transiently expressing TLR3 following 6 h of treatment with 1 µg/mL poly(I:C). Data are presented as mean ± SD (*n* = 3). ****P < 0.0001, one-way ANOVA with Bonferroni’s post hoc test. (*B*) Western blot analysis of cell lysates from *A*. (*C*) WT and GIGYF2-KO HEK293 cells were transfected with the plasmids expressing v5-empty or v5-4EHP. ELISA measurement of IFN-β production was performed following 6 h of 1 µg/mL High molecular weight (HMW) poly(I:C) treatment. Data are presented as mean ± SD (*n* = 3). ns, nonsignificant, two-tailed Student’s *t* test. (*D*) Western blot analysis of lysates from *C*. (*E*) v5-empty or v5-4EHP expression plasmids were cotransfected with EV or plasmids expressing WT GIGYF2 or 4EHP-binding mutant GIGYF2 (Y41A, Y43A, M48A, L49A; GIGYF2M). IFN-β ELISA was performed following 6 h of 1 µg/mL HMW poly(I:C) stimulation. Data are presented as mean ± SD (*n* = 3). ***P < 0.001, one-way ANOVA with Bonferroni’s post hoc test. (*F*) Western blot analysis of lysates from *D*. (*G*) WT, 4EHP-KO, GIGYF1-KO, and GIGYF2-KO HEK293 cells were transfected with psiCHECK2-*R-Luc*-*Ifnb1* 3′ UTR reporter or the psiCHECK2 reporter (as control). *R-Luc* and *F-Luc* activities were measured 24 h after transfection. The *R-Luc*/*F-Luc* ratio in psiCHECK2-*R-Luc*-*Ifnb1* 3′ UTR reporter–expressing cells was normalized to the psiCHECK2-expressing cells. Data are presented as mean ± SD (*n* = 3). ns, nonsignificant, **P < 0.01, ***P < 0.001, two-way ANOVA with Bonferroni’s post hoc test. (*H–K*) WT or GIGYF2-KO#1–2 (two independent sgRNAs) A549 cells were infected with mock or VSVΔ51-GFP (0.1 multiplicity of infection [MOI]). At 12 h postinfection (h.p.i.), cells were subjected to: (*H*) visualization of VSVΔ51-GFP infection by fluorescence microscopy, (*I*) quantification of the GFP-positive cell number in *F* by ImageJ, and (*J*) ELISA measurement of IFN-β production in the supernatant. (*K*) At the indicated time points post virus infection, *ISG56* mRNA levels were measured by RT-qPCR. *GAPDH* mRNA was used for normalization. Data are presented as mean ± SD (*n* = 5). **P < 0.01, ***P < 0.001, ****P < 0.0001, one-way ANOVA with Bonferroni’s post hoc test. See also *SI Appendix*, Figs. S4 and S5.

We reported that 4EHP suppresses *Ifnb1* mRNA translation (25). 4EHP interacts with GIGYF2 and mediates GIGYF2-induced translational repression (23, 30). Thus, we examined the role of GIGYF2 in the regulation of IFN-β production. Due to the lack of expression of Toll-like receptor 3 (TLR3) in HEK293 cells, TLR3 was transiently expressed in WT, 4EHP-KO, and GIGYF2-KO HEK293 cells, which were then treated with poly(I:C), an agonist of TLR3 that stimulates IFN-β production. While, as expected (25), 4EHP-KO cells produced ∼2.5-fold more IFN-β than WT cells, a significantly more robust (∼5.5-fold) increase in IFN-β production was observed in GIGYF2-KO, compared with WT cells (Fig. 3*A*). Furthermore, consistent with the elevated IFN-β production, poly(I:C)-induced STAT1 phosphorylation was enhanced (∼3-fold) in 4EHP-KO HEK293 cells, an effect which was further augmented (∼6.5-fold) in GIGYF2-KO cells (Fig. 3*B*).

Next, we examined whether GIGYF2 also suppresses IFN-β production in two lung epithelial cell lines, Calu-3 and A549, which are widely used in SARS-CoV-2 studies and respond to poly(I:C) stimulation with robust IFN-β production (34–37). Upon poly(I:C) treatment of Calu-3 and A549 cells, IFN-β expression and STAT1 phosphorylation significantly increased in the 4EHP-depleted cells (∼2.5-fold in Calu-3 and ∼1.8-fold in A549 cells), and even more in GIGYF2-depleted cells compared to WT cells (∼6-fold in Calu-3 and ∼6.1-fold in A549 cells; *SI Appendix*, Fig. S4 *A*–*D*). These data demonstrate that 4EHP and GIGYF2 repress IFN-β production and that GIGYF2 is a more potent repressor than 4EHP. Importantly, *Ifnb1* mRNA levels did not change in 4EHP- or GIGYF2-depleted cells compared to their control counterparts (*SI Appendix*, Fig. S4 *E*–*G*).

Next, we investigated whether formation of the GIGYF2/4EHP complex is necessary for repression of IFN-β production. Rescuing expression of 4EHP, which is destabilized in GIGYF2-depleted cells (20), failed to restore repression of IFN-β production in GIGYF2-KO HEK293 cells (Fig. 3 *C* and *D*). These data indicate that repression of IFN-β production by 4EHP is dependent on GIGYF2. To determine whether direct interaction of 4EHP with GIGYF2 is required for repression of IFN-β production, we overexpressed 4EHP and WT GIGYF2 or a mutant form of GIGYF2 (GIGYF2M), which does not bind to 4EHP (20), in GIGYF2-KO cells. Coexpression of 4EHP and WT GIGYF2 restored the repression of IFN-β in GIGYF2-KO cells (Fig. 3 *E* and *F*). In contrast, cotransfection of 4EHP and GIGYF2M only partially (∼50%) rescued the IFN-β repression, indicating that formation of the GIGYF2/4EHP complex is pivotal for efficient repression of IFN-β production.

The 3′ UTR of the *Ifnb1* mRNA plays a key role in 4EHP-mediated translational repression (25). To investigate whether the *Ifnb1* mRNA 3′ UTR exerts its silencing effect via GIGYF2, we transfected a luciferase reporter (*R-Luc*) fused to the *Ifnb1* 3′ UTR into WT, 4EHP-KO, GIGYF1-KO, or GIGYF2-KO cells. Luciferase activity was repressed by twofold in WT and GIGYF1-KO cells, but not in 4EHP-KO and GIGYF2-KO cells (Fig. 3*G*), with no change in the abundance of the mRNA (*SI Appendix*, Fig. S5*A*). These data demonstrate that GIGYF2 and 4EHP mediate the translational silencing induced by *Ifnb1* mRNA 3′ UTR.

### GIGYF2 Represses RNA Virus Replication.

To assess the broader role of GIGYF2 in the antiviral immune response to RNA viruses through repression of IFN-β production, we used a GFP-tagged mutant strain of vesicular stomatitis virus (VSVΔ51). The deletion of methionine-51 (M51) in the matrix protein, renders the virus more sensitive to the IFN antiviral activity (38). We reported that 4EHP depletion inhibits the replication of VSVΔ51 by enhancing the production of IFN-β (25). GIGYF2-KO significantly limited replication of GFP-tagged VSVΔ51 in A549 lung cells 12 h postinfection (Fig. 3 *H* and *I*). Following virus infection, expression of IFN-β (measured by enzyme-linked immunosorbent assay [ELISA]) and the mRNA level of the IFN-stimulated gene 56 (ISG56, measured by RT-qPCR) were increased approximately twofold as compared to WT cells (Fig. 3 *J* and *K*) without a detectable change in *Ifnb1* mRNA levels (*SI Appendix*, Fig. S5*B*). These data support the conclusion that GIGYF2-depletion protects A549 cells from VSVΔ51-GFP infection, due to robust IFN-β production and activation of IFN-induced antiviral pathways.

We next asked whether GIGYF2 directly targets virus-induced activation of signaling pathways upstream of IFN-β. We first examined the impact of GIGYF2 or 4EHP depletion on virus RNA sensor–initiated signaling. We cotransfected GIGYF2-KO cells with a *F-Luc* reporter under the control of the minimal IFN-β promoter and constructs expressing constitutively active forms of key factors involved in RNA virus–induced signaling to mimic TLR3- and retinoic acid inducible gene I (RIG-I)-like receptor (RLR)-mediated signaling. GIGYF2 depletion did not affect IFN-β promoter activity mediated by upstream signaling pathways (*SI Appendix*, Fig. S5*C*). We also examined whether GIGYF2 depletion affects JAK-STAT, a key downstream signaling pathway activated by IFN-β. We transfected the *F-Luc* reporter under the control of an IFN-sensitive response element (ISRE) promoter into WT, 4EHP-KO, or GIGYF2-KO HEK293 cells, followed by treatment with increasing doses of recombinant IFN-β protein. ISRE reporter activity was not affected by the removal of GIGYF2 or 4EHP-KO (*SI Appendix*, Fig. S5*D*). Neither did the removal of 4EHP or GIGYF2 affect recombinant IFN-β–induced STAT1 phosphorylation or ISG56 expression compared with WT cells (*SI Appendix*, Fig. S5*E*). These results demonstrate that the GIGYF2- and 4EHP-mediated antiviral immune response is a consequence of direct repression of *Ifnb1* mRNA translation and not via directly affecting the RNA virus sensors or signaling pathways downstream of IFN-β.

### SARS-CoV-2 NSP2 Coopts the GIGYF2/4EHP Complex to Repress *Ifnb1* mRNA Translation and Facilitate SARS-CoV-2 Replication.

**Fig. 4.**
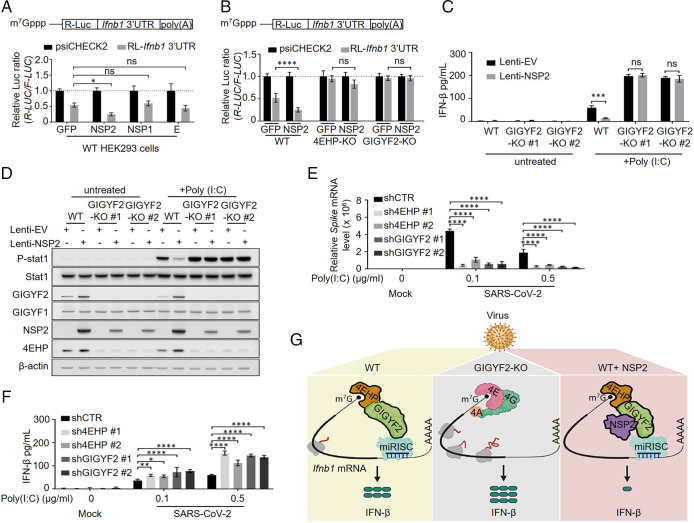
NSP2 augments the GIGYF2/4EHP complex–mediated translational silencing of *Ifnb1* mRNA. (*A*) The 24 h posttransfection with GFP, NSP2, NSP1, or E protein, HEK293 cells were transfected with psiCHECK2-*R-Luc-Ifnb1* 3′ UTR reporter or the psiCHECK2 reporter (control). *R-Luc* and *F-Luc* activities were measured 24 h after the second transfection. The *R-Luc/F-Luc* ratio of psiCHECK2-*R-Luc-Ifnb1* 3′ UTR cells was normalized to the value for the psiCHECK2 cells as a percentage. Data are presented as mean ± SD (*n* = 3). ns, nonsignificant, *P < 0.05, two-way ANOVA with Bonferroni’s post hoc test. (*B*) WT, 4EHP-KO, and GIGYF2-KO HEK293 cells were transfected with GFP or NSP2 expression plasmid for 24 h, followed by the second transfection with psiCHECK2-*R-Luc*-*Ifnb1* 3′ UTR reporter or the psiCHECK2 control reporter. At 24 h posttransfection the *R-Luc*/*F-Luc* ratio was measured as described in *A*. (*C*) ELISA measurement of IFN-β and (*D*) Western blot analysis of 1 µg/mL poly(I:C)-treated WT and GIGYF2-KO A549 cells stably expressing either EV or NSP2 using lentiviral vector at 8 h posttransfection. (*E*) shCTR, sh4EHP, and shGIGYF2 Calu-3 cells were challenged with 0.1 or 0.5 µg/mL poly(I:C) for 6 h, followed by 0.01 MOI SARS-CoV2 infection, or mock-infected group as control. At 24 h postinfection, viral mRNA levels were measured by RT-qPCR, normalized to the *GAPDH* mRNA, and expressed as fold values relative to the mock-infected shCTR cells. (*F*) The cell culture supernatants collected from the samples described in *E* were used for detection of IFN-β levels with ELISA. Data are presented as mean ± SD (*n* = 3). ns, nonsignificant, *P < 0.05, **P < 0.01, ***P < 0.001, ****P < 0.0001; two-way ANOVA with Bonferroni’s post hoc test was used. (*G*) Graphic illustration of cooption of the GIGYF2/4EHP repressor complex by NSP2 to silence IFN-β production in response to SARS-CoV-2 infection. The GIGYF2/4EHP complex enables the miRISC-induced repression of the cap-dependent mRNA translation. Binding of NSP2 to GIGYF2 enhances the interaction of GIGYF2 with 4EHP, resulting in costabilization of GIGYF2 and 4EHP and augmented translational repression of *Ifnb1* mRNA. See also *SI Appendix*, Figs. S6 and S7.

To investigate the potential role of NSP2 in the control of IFN-β expression, SARS-CoV-2 NSP2, NSP1, or envelope (E) protein were expressed in HEK293 cells cotransfected with the *R-Luc* reporter construct fused to the *Ifnb1* 3′ UTR. The reporter expression was repressed approximately twofold upon ectopic expression of NSP2, but not NSP1 or E protein (Fig. 4*A* and *SI Appendix*, Fig. S6*A*). To examine whether NSP2-mediated repression of the *R-Luc*-*Ifnb1* 3′ UTR reporter requires the GIGYF2/4EHP complex, reporter activity was measured in WT, 4EHP-KO, and GIGYF2-KO cells expressing either GFP or NSP2 (*SI Appendix*, Fig. S6*B*). *R-Luc*-*Ifnb1* 3′ UTR was consistently repressed by twofold in the NSP2-transfected WT cells compared to the GFP-transfected WT cells (Fig. 4*B*), without affecting mRNA levels (*SI Appendix*, Fig. S6*C*). However, silencing of the *R-Luc*-*Ifnb1* 3′ UTR reporter was relieved in 4EHP-KO and GIGYF2-KO cells, regardless of the expression of NSP2 (Fig. 4*B*). Thus, the NSP2-induced *Ifnb1* 3′ UTR–dependent repression requires the presence of 4EHP and GIGYF2. Notably, transient expression of NSP2 in WT HEK293 cells elicited an ∼40% and ∼48% increase in endogenous GIGYF2 and 4EHP protein levels, respectively (*SI Appendix*, Fig. S6 *B*, *D*, and *E*), without affecting GIGYF2 and 4EHP mRNA levels (*SI Appendix*, Fig. S6 *F* and *G*). This is likely due to the NSP2-induced enhanced GIGYF2 and 4EHP interaction (Fig. 2), which engenders mutual stabilization of both proteins (20).

Next, we wished to confirm the above results in the lung epithelial cell line A549. We used lentiviral vectors to stably express NSP2 or control empty vector (EV) in WT and GIGYF2-KO A549 cells. Compared to control, ectopic NSP2 expression reduced the poly(I:C)-induced IFN-β production (∼4-fold; Fig. 4*C*) and STAT1 phosphorylation (∼4-fold; Fig. 4*D*) in WT cells, without a significant impact on *Ifnb1* mRNA levels (*SI Appendix*, Fig. S7*A*). In stark contrast, NSP2 failed to repress IFN-β production or STAT1 phosphorylation in GIGYF2-KO cells (Fig. 4 *C* and *D*). Notably, similar to HEK293T cells (*SI Appendix*, Fig. S6*B*), stable expression of NSP2 in A549 cells resulted in a 2-fold and 1.8-fold increase in GIGYF2 and 4EHP protein levels, respectively (Fig. 4*D* and *SI Appendix*, Fig. S7 *D* and *E*), without changes in mRNA levels (*SI Appendix*, Fig. S7 *B* and *C*). However, stabilization of 4EHP by NSP2 was not observed in NSP2-expressing GIGYF2-KO cells, because NSP2 directly interacts with GIGYF2, but not 4EHP.

We next investigated whether the GIGYF2/4EHP-mediated repression of IFN-β production affects SARS-CoV-2 replication. Since SARS-CoV-2 significantly represses the expression of *Ifnb1* mRNA in Calu3 cells (10, 11), we pretreated the cells with poly(I:C) to bolster the IFN-β secretion before SARS-CoV-2 exposure (Fig. 4*E*). Following 6 h of pretreatment with poly(I:C), control (shCTR), 4EHP-knockdown (sh4EHP#1–2), and GIGYF2-knockdown (shGIGYF2#1–2) Calu-3 cells were infected with SARS-CoV-2. After 24 h of infection, we assessed the viral RNA levels to evaluate the virus replication status using RT-qPCR, and measured IFN-β production by ELISA. As expected, both sh4EHP- and shGIGYF2-treated cells followed by mock infection expressed more IFN-β compared to shCTR cells (*SI Appendix*, Fig. S4*A*). Importantly, upon pretreatment with 0.1 or 0.5 µg/mL poly(I:C), sh4EHP and shGIGYF2 reduced spike mRNA levels by 70% and 80%, respectively, compared to shCTR (Fig. 4*E*). A similar result was observed for the SARS-CoV-2 nucleocapsid (N) protein mRNA by RT-qPCR (*SI Appendix*, Fig. S7*F*). Consistently, the IFN-β levels in the supernatant were significantly higher in sh4EHP and shGIGYF2 cells compared to shCTR cells upon SARS-CoV-2 infection [respectively, ∼50% and ∼95% increase in 0.1 µg/mL poly(I:C) pretreated cells and ∼100% and ∼135% increase in 0.5 µg/mL poly(I:C) pretreated cells; Fig. 4*F*].

Taken together, our data offer a mechanistic model in which NSP2 directly interacts with host GIGYF2 protein to enhance the interaction of GIGYF2 with 4EHP, resulting in stabilization of both GIGYF2 and 4EHP proteins. We show that NSP2 coopts the GIGYF2/4EHP translational repression complex to suppress IFN-β production and thereby facilitates viral replication (model; Fig. 4*G*).

## Discussion

While a robust type I IFN-mediated antiviral innate immune response is indispensable for combating infections, an exacerbated response can result in pathological inflammation and tissue damage (39–42). mRNA translational control mechanisms play a crucial role in maintaining the appropriate magnitude and duration of the immune response (42). Our data show that the GIGYF2/4EHP complex inhibits translation of *Ifnb1* mRNA. We demonstrate that SARS-CoV-2 coopts this mechanism through NSP2, which is highly conserved among coronaviruses (28) to impede the antiviral innate immune response.

Notably, the interaction of SARS-CoV-1 encoded NSP2 with GIGYF2 and 4EHP was also reported (43), indicating a common mechanism of impeding the host innate immune response by coronaviruses. Our data reveal that another RNA virus (VSV) also employs the GIGYF2/4EHP complex to repress IFN-β production (Fig. 3) (25). Thus, it appears that at least two different RNA viruses use distinct means that converge on GIGYF2/4EHP translational repression complex to block the activation of the antiviral innate immune response. A recent study showed that SARS-CoV-2 expresses a miRNA-like small RNA that selectively repress host genes related to activation of interferon signaling (44). It is possible that SARS-CoV-2–encoded NSP2 could also enhance the repression of the cellular targets of the viral coded miRNAs via coopting the GIGYF2/4EHP complex.

Other SARS-CoV-2 proteins, including NSP1 and NSP14, also dysregulate the host mRNA translation machinery (13, 16, 22, 43, 45). NSP1 blocks the ribosomal entry site for host mRNAs but allows SARS-CoV-2 mRNA translation (46, 47). While viral RNA is protected, host mRNA is subjected to degradation. Thus, NSP1 broadly inhibits translation of host mRNAs, including *Ifnb1* (47), and results in depletion of antiviral factors such as Tyk2 and STAT2 (48). NSP14 also inhibits global mRNA translation, which likewise involves the shutdown of ISG expression (45). In contrast, we showed that NSP2 associates with the GIGYF2/4EHP complex to repress translation of *Ifnb1* mRNA, but it is highly likely that this mechanism also affects the expression of other important cytokines that promote an antiviral response.

The N-terminal region of GIGYF2 encodes several important protein binding motifs, including the 4EHP-binding motif (20), DDX6-binding motif, and the GYF domain that interacts with the Pro-Pro-Gly-hydrophobic motif (PPGL) (20, 49). We mapped an NSP2 binding region at the LHR of GIGYF2. The solution of the three-dimensional structure of the LHR and its interaction with NSP2 will be instrumental for a better understanding of the molecular basis of the proposed NSP2/GIGYF2/4EHP complex. The knowledge of the mechanism of action of NSP2-mediated IFN suppression via the 4EHP/GIGYF2 complex and identifying the binding motif on NSP2 and GIGYF2 LHR could inform the development of peptides or small molecules to block the interaction of NSP2 with GIGYF2. These findings have potentially considerable value for combatting future infections of SARS-CoV-2 and of other known and yet to emerge novel coronaviruses.

## Methods

### Cell Lines and Culture Conditions.

HEK293T (Thermo Fisher Scientific) cells were cultured in Dulbecco’s modified Eagle medium (DMEM) supplemented with 10% fetal bovine serum (FBS) and 1% penicillin/streptomycin (P/S) (Wisent Technologies). A549 (American Type Culture Collection [ATCC]), were cultured in RPMI, also supplemented with 10% FBS and 1% P/S. Calu-3 cells (ATCC) were cultured in Eagle minimal essential medium (EMEM) supplemented with 20% FBS and 1% P/S. WT, 4EHP-knockout, and GIGYF2-knockout HEK293 cells were maintained in DMEM supplemented with 10% FBS, 1% P/S, 100 µg/mL zeocin (Thermo Fisher Scientific, R25001), and 15 µg/mL blasticidin (Thermo Fisher Scientific, R210-01) (17). All cells were cultured at 37 °C, in a humidified atmosphere with 5% CO_2_.

### PLA.

PLA using Duolink reagents (Sigma, DUO92101) was performed according to the manufacturer’s recommendations. Briefly, cells were fixed with 4% PFA-sucrose Paraformaldehyde (PFA) for 15 min and permeabilized by Phosphate-buffered saline (PBS) containing 0.1% Triton X-100 for 15 min. Cells were blocked in Duolink blocking solution for 1 h at 37 °C and incubated with primary antibodies overnight at 4 °C. Cells were washed by wash buffer A before incubation with PLA probe for 1 h at 37 °C, followed by ligation for 30 min at 37 °C. PLA signals were amplified using amplification buffer for 100 min at 37 °C, followed by washing with wash buffer B and mounting onto the glass slide before Airyscan microscopic imaging (Zeiss). Further information is given in *SI Appendix*, *SI Materials and Methods*.

## Supplementary Material

Supplementary File

## Data Availability

All study data are included in the article and/or supporting information.

## References

[1] F. McNab, K. Mayer-Barber, A. Sher, A. Wack, A. O’Garra, Type I interferons in infectious disease. Nat. Rev. Immunol. 15, 87–103 (2015). 25614319 10.1038/nri3787 PMC7162685

[2] U. Felgenhauer , Inhibition of SARS-CoV-2 by type I and type III interferons. J. Biol. Chem. 295, 13958–13964 (2020). 32587093 10.1074/jbc.AC120.013788 PMC7549028

[3] K. G. Lokugamage , Type I Interferon Susceptibility Distinguishes SARS-CoV-2 from SARS-CoV. J. Virol. 94, e01410-20 (2020). 10.1128/JVI.01410-20 PMC7654262 32938761

[4] E. Mantlo, N. Bukreyeva, J. Maruyama, S. Paessler, C. Huang, Antiviral activities of type I interferons to SARS-CoV-2 infection. Antiviral Res. 179, 104811 (2020). 32360182 10.1016/j.antiviral.2020.104811 PMC7188648

[5] P. Bastard ; HGID Lab; NIAID-USUHS Immune Response to COVID Group; COVID Clinicians; COVID-STORM Clinicians; Imagine COVID Group; French COVID Cohort Study Group; Milieu Intérieur Consortium; CoV-Contact Cohort; Amsterdam UMC Covid-19 Biobank; COVID Human Genetic Effort, Autoantibodies against type I IFNs in patients with life-threatening COVID-19. Science 370, eabd4585 (2020). 32972996 10.1126/science.abd4585 PMC7857397

[6] Q. Zhang ; COVID-STORM Clinicians; COVID Clinicians; Imagine COVID Group; French COVID Cohort Study Group; CoV-Contact Cohort; Amsterdam UMC Covid-19 Biobank; COVID Human Genetic Effort; NIAID-USUHS/TAGC COVID Immunity Group, Inborn errors of type I IFN immunity in patients with life-threatening COVID-19. Science 370, eabd4570 (2020). 32972995 10.1126/science.abd4570 PMC7857407

[7] K. I. Masood , Upregulated type I interferon responses in asymptomatic COVID-19 infection are associated with improved clinical outcome. Sci. Rep. 11, 22958 (2021). 34824360 10.1038/s41598-021-02489-4 PMC8617268

[8] D. C. Vinh ; COVID Human Genetic Effort, Harnessing type I IFN immunity against SARS-CoV-2 with early administration of IFN-β. J. Clin. Immunol. 41, 1425–1442 (2021). 34101091 10.1007/s10875-021-01068-6 PMC8186356

[9] J. Hadjadj , Impaired type I interferon activity and inflammatory responses in severe COVID-19 patients. Science 369, 718–724 (2020). 32661059 10.1126/science.abc6027 PMC7402632

[10] M. Shemesh , SARS-CoV-2 suppresses IFNβ production mediated by NSP1, 5, 6, 15, ORF6 and ORF7b but does not suppress the effects of added interferon. PLoS Pathog. 17, e1009800 (2021). 34437657 10.1371/journal.ppat.1009800 PMC8389490

[11] X. Lei , Activation and evasion of type I interferon responses by SARS-CoV-2. Nat. Commun. 11, 3810 (2020). 32733001 10.1038/s41467-020-17665-9 PMC7392898

[12] M. R. Alexander , Ribosome-profiling reveals restricted post transcriptional expression of antiviral cytokines and transcription factors during SARS-CoV-2 infection. Int. J. Mol. Sci. 22, 3392 (2021). 33806254 10.3390/ijms22073392 PMC8036502

[13] Y. Finkel , SARS-CoV-2 uses a multipronged strategy to impede host protein synthesis. Nature 594, 240–245 (2021). 33979833 10.1038/s41586-021-03610-3

[14] R. K. Suryawanshi, R. Koganti, A. Agelidis, C. D. Patil, D. Shukla, Dysregulation of Cell Signaling by SARS-CoV-2. Trends Microbiol. 29, 224–237 (2021). 33451855 10.1016/j.tim.2020.12.007 PMC7836829

[15] N. Sonenberg, A. G. Hinnebusch, Regulation of translation initiation in eukaryotes: mechanisms and biological targets. Cell 136, 731–745 (2009). 19239892 10.1016/j.cell.2009.01.042 PMC3610329

[16] M. Christie, C. Igreja, eIF4E-homologous protein (4EHP): a multifarious cap-binding protein. FEBS J. 10.1111/febs.16275 (2021). 10.1111/febs.16275 34758096

[17] S. M. Jafarnejad , Translational control of ERK signaling through miRNA/4EHP-directed silencing. eLife 7, e35034 (2018). 29412140 10.7554/eLife.35034 PMC5819943

[18] C. Chapat , Cap-binding protein 4EHP effects translation silencing by microRNAs. Proc. Natl. Acad. Sci. U.S.A. 114, 5425–5430 (2017). 28487484 10.1073/pnas.1701488114 PMC5448183

[19] V. K. Mayya , microRNA-mediated translation repression through GYF-1 and IFE-4 in C. elegans development. Nucleic Acids Res. 49, 4803–4815 (2021). 33758928 10.1093/nar/gkab162 PMC8136787

[20] M. Morita , A novel 4EHP-GIGYF2 translational repressor complex is essential for mammalian development. Mol. Cell. Biol. 32, 3585–3593 (2012). 22751931 10.1128/MCB.00455-12 PMC3422012

[21] S. Juszkiewicz , Ribosome collisions trigger cis-acting feedback inhibition of translation initiation. eLife 9, 9 (2020). 10.7554/eLife.60038 PMC7381030 32657267

[22] K. L. Hickey , GIGYF2 and 4EHP inhibit translation initiation of defective messenger RNAs to assist ribosome-associated quality control. Mol. Cell 79, 950–962.e6 (2020). 32726578 10.1016/j.molcel.2020.07.007 PMC7891188

[23] C. C. Amaya Ramirez, P. Hubbe, N. Mandel, J. Béthune, 4EHP-independent repression of endogenous mRNAs by the RNA-binding protein GIGYF2. Nucleic Acids Res. 46, 5792–5808 (2018). 29554310 10.1093/nar/gky198 PMC6009589

[24] R. Fu, M. T. Olsen, K. Webb, E. J. Bennett, J. Lykke-Andersen, Recruitment of the 4EHP-GYF2 cap-binding complex to tetraproline motifs of tristetraprolin promotes repression and degradation of mRNAs with AU-rich elements. RNA 22, 373–382 (2016). 26763119 10.1261/rna.054833.115 PMC4748815

[25] X. Zhang , microRNA-induced translational control of antiviral immunity by the cap-binding protein 4EHP. Mol. Cell 81, 1187–1199.e5 (2021). 33581076 10.1016/j.molcel.2021.01.030

[26] J. P. Davies, K. M. Almasy, E. F. McDonald, L. Plate, Comparative multiplexed interactomics of SARS-CoV-2 and homologous coronavirus nonstructural proteins identifies unique and shared host-cell dependencies. ACS Infect. Dis. 6, 3174–3189 (2020). 33263384 10.1021/acsinfecdis.0c00500 PMC7724760

[27] D. E. Gordon , A SARS-CoV-2 protein interaction map reveals targets for drug repurposing. Nature 583, 459–468 (2020). 32353859 10.1038/s41586-020-2286-9 PMC7431030

[28] M. Gupta , CryoEM and AI reveal a structure of SARS-CoV-2 Nsp2, a multifunctional protein involved in key host processes. bioRxiv. [Preprint] (2021). 10.1101/2021.05.10.443524. Accessed 19 July 2022.

[29] J. Jumper , Highly accurate protein structure prediction with AlphaFold. Nature 596, 583–589 (2021). 34265844 10.1038/s41586-021-03819-2 PMC8371605

[30] D. Peter , GIGYF1/2 proteins use auxiliary sequences to selectively bind to 4EHP and repress target mRNA expression. Genes Dev. 31, 1147–1161 (2017). 28698298 10.1101/gad.299420.117 PMC5538437

[31] T. Fukaya, Y. Tomari, MicroRNAs mediate gene silencing via multiple different pathways in drosophila. Mol. Cell 48, 825–836 (2012). 23123195 10.1016/j.molcel.2012.09.024

[32] J. Baron-Benhamou, N. H. Gehring, A. E. Kulozik, M. W. Hentze, Using the lambdaN peptide to tether proteins to RNAs. Methods Mol. Biol. 257, 135–154 (2004). 14770003 10.1385/1-59259-750-5:135

[33] J. Zhao, X. Zhai, J. Zhou, (2020) Snapshot of the evolution and mutation patterns of SARS-CoV-2. *bioRxiv*. doi: 10.1101/2020.07.04.187435 (2020).

[34] V. Cagno, SARS-CoV-2 cellular tropism. Lancet Microbe 1, e2–e3 (2020). 32835319 10.1016/S2666-5247(20)30008-2 PMC7173832

[35] A. Rebendenne , SARS-CoV-2 triggers an MDA-5-dependent interferon response which is unable to control replication in lung epithelial cells. J. Virol. 10.1128/jvi.02415-20 (2021). PMC8103705 33514628

[36] A. C. Hsu , Critical role of constitutive type I interferon response in bronchial epithelial cell to influenza infection. PLoS One 7, e32947 (2012). 22396801 10.1371/journal.pone.0032947 PMC3292582

[37] M. L. Stanifer , Critical Role of Type III Interferon in Controlling SARS-CoV-2 Infection in Human Intestinal Epithelial Cells. Cell Rep. 32, 107863 (2020). 32610043 10.1016/j.celrep.2020.107863 PMC7303637

[38] X. Lun , Effects of intravenously administered recombinant vesicular stomatitis virus (VSV(deltaM51)) on multifocal and invasive gliomas. J. Natl. Cancer Inst. 98, 1546–1557 (2006). 17077357 10.1093/jnci/djj413

[39] P. Anderson, Post-transcriptional control of cytokine production. Nat. Immunol. 9, 353–359 (2008). 18349815 10.1038/ni1584

[40] J. Banchereau, V. Pascual, Type I interferon in systemic lupus erythematosus and other autoimmune diseases. Immunity 25, 383–392 (2006). 16979570 10.1016/j.immuni.2006.08.010

[41] T. Mino, O. Takeuchi, Post-transcriptional regulation of immune responses by RNA binding proteins. Proc. Jpn. Acad., Ser. B, Phys. Biol. Sci. 94, 248–258 (2018). 10.2183/pjab.94.017 PMC6085518 29887569

[42] S. Carpenter, E. P. Ricci, B. C. Mercier, M. J. Moore, K. A. Fitzgerald, Post-transcriptional regulation of gene expression in innate immunity. Nat. Rev. Immunol. 14, 361–376 (2014). 24854588 10.1038/nri3682

[43] C. T. Cornillez-Ty, L. Liao, J. R. Yates, 3rd, P. Kuhn, M. J. Buchmeier, Severe acute respiratory syndrome coronavirus nonstructural protein 2 interacts with a host protein complex involved in mitochondrial biogenesis and intracellular signaling. J. Virol. 83, 10314–10318 (2009). 19640993 10.1128/JVI.00842-09 PMC2748024

[44] P. Pawlica , SARS-CoV-2 expresses a microRNA-like small RNA able to selectively repress host genes. Proc. Natl. Acad. Sci. U.S.A. 118, e2116668118 (2021). 34903581 10.1073/pnas.2116668118 PMC8719879

[45] J. C. Hsu, M. Laurent-Rolle, J. B. Pawlak, C. B. Wilen, P. Cresswell, Translational shutdown and evasion of the innate immune response by SARS-CoV-2 NSP14 protein. Proc. Natl. Acad. Sci. U.S.A. 118, e2101161118 (2021). 34045361 10.1073/pnas.2101161118 PMC8214666

[46] K. Schubert , SARS-CoV-2 Nsp1 binds the ribosomal mRNA channel to inhibit translation. Nat. Struct. Mol. Biol. 27, 959–966 (2020). 32908316 10.1038/s41594-020-0511-8

[47] M. Thoms , Structural basis for translational shutdown and immune evasion by the Nsp1 protein of SARS-CoV-2. Science 369, 1249–1255 (2020). 32680882 10.1126/science.abc8665 PMC7402621

[48] A. Kumar , SARS-CoV-2 Nonstructural Protein 1 Inhibits the Interferon Response by Causing Depletion of Key Host Signaling Factors. J. Virol. 95, e0026621 (2021). 34110264 10.1128/JVI.00266-21 PMC8316110

[49] D. Peter , Molecular basis for GIGYF-Me31B complex assembly in 4EHP-mediated translational repression. Genes Dev. 33, 1355–1360 (2019). 31439631 10.1101/gad.329219.119 PMC6771390

