# Supplementary material for: SARS-CoV-2 impairs interferon production via NSP2-induced repression of mRNA translation
Source: Proc Natl Acad Sci U S A. 2022 Jul 25;119(32):e2204539119. doi: 10.1073/pnas.2204539119 (PMC9371684; doi:10.1073/pnas.2204539119)
Supplement: Supplementary File [file pnas.2204539119.sapp.pdf]

PNAS
www.pnas.org

Supplementary Information for

SARS-CoV-2 impairs interferon production via NSP2-induced repression of mRNA translation

Xu Zhang*, Jung-Hyun Choi*, David L. Dai, Jun Luo, Reese Jalal Ladak, Qian Li, Yimeng Wang, Christine Zhang, Shane Wiebe, Alex C.H. Liu, Xiaozhuo Ran, Jiaqi Yang, Parisa Naeli, Aitor Garzia, Lele Zhou, Niaz Mahmood, Qiyun Deng, Mohamed Elaish, Rongtuan Lin, Lara K. Mahal, Tom C. Hobman, Jerry Pelletier, Tommy Alain, Silvia M. Vidal, Thomas Duchaine, Mohammad T. Mazhab-Jafari, Xiaojuan Mao, Seyed Mehdi Jafarnejad#, and Nahum Sonenberg#

*These authors contributed equally to this work
#Correspondence should be addressed to N.S.: (nahum.sonenberg@mcgill.ca) and S.M.J. (sm.jafarnejad@qub.ac.uk).

This PDF file includes:

Supplementary text
Figures S1 to S7
Tables S1

Supplementary Information Text

SI Materials and Mathods

Antibodies, siRNAs, shRNAs, and plasmids

The following antibodies were used: rabbit anti-eIF4E2 (Genetex, GTX103977 and GTX64395), rabbit anti-GIGYF1 (Bethyl Laboratories, A304-132A), rabbit anti-GIGYF2 (Bethyl Laboratories, A303-732A), sheep anti-SARS-CoV-2-NSP2 (MRC PPU reagents and Services, DA105), mouse anti-β-actin (Sigma, A5441), rabbit anti-STAT1 (Cell Signaling, 14994), rabbit anti-phospho-STAT1 (Tyr701; Cell signaling, 7649), rabbit anti-TLR3 (Cell signaling, 6961), rabbit anti-v5 (abcam, ab9116), mouse anti-FLAG (abcam, ab49763). The following shRNAs were used: Non-Targeting Control shRNA (Sigma, SHC002), EIF4E2 shRNA#1 (sh4EHP#1) (Sigma, TRCN0000152006), EIF4E2 shRNA#2 (sh4EHP#2) (Sigma, TRCN0000280916) and EIF4E2 shRNA#3 (sh4EHP#3) (Sigma,). GIGYF2 shRNA#1 (shGIGYF2#1) (Sigma, TRCN0000135151), GIGYF2 shRNA#2 (shGIGYF2#2) (Sigma, TRCN0000138937) and GIGYF2 shRNA#3 (shGIGYF2#3) (Sigma, TRCN0000135088).

Plasmids encoding *Firefly luciferase* (*F-Luc*) driven by either the ISRE promoter (ISRE-Luc) or the *Ifnb1* promoter (IFN-β–Luc) were used for the reporter assay. The pRL-TK vector (Promega, E2241) encoding *Renilla luciferase* (*R-Luc*) was used as a transfection control. TLR-3 expressing plasmid was used to transfect HEK293 cells transiently. The plasmids encoding human RIG-I, MAVS, TBK1, TRIF, and IRF3 have been previously described(56). The pLenti-CMV-GFP-Puro (Addgene, 17448), pLenti-CMV-Luc-Puro (Addgene, 17477), Lenti-4EHP (Sigma, TRCN0000474313), and pLenti-X2-Zeo-DEST (Addgene, 21562) were used to generate cells which stably express GFP, luciferase,4EHP, and NSP2, respectively. The Lenti-Cas9-Blast (Addgene, plasmid 52962) and pLenti-CRISPRv2 (Addgene, plasmid 52961) were used to generate the A549 4EHP and GIGYF2 knockout.

Generation of knockout cell lines by CRISPR-Cas9. CRISPR-Cas9-mediated genome editing of Flp-In t-REx HEK293 cells was performed as previously described(57). The oligodeoxynucleotides encoding sgRNAs for targeting the coding region of the gene of interest are listed in SI Appendix 8. Briefly, the forward and reverse strand oligodeoxynucleotides were annealed and ligated into pSpCas9(BB)-2A-GFP (Addgene, PX458, Plasmid #48138) linearized with BbsI (Thermo Fisher Scientific, ER1011). After transformation, the guide sequence containing pSpCas9(BB)2A-GFP plasmids were isolated and sequence-verified. To generate gene knockout Flp-In t-REx HEK293 cells, 130,000 cells were transfected with the corresponding guide sequence containing pSpCas9(BB)-2A-GFP plasmid. 24 h after transfection, GFP positive cells were-single cell sorted by FACS into two 96-well plates and cultivated until colonies were obtained.

The 4EHP-KO and GIGYF2-KO A549 cells were generated in two steps. Firstly, the A549 lenti-Cas9 cells were generated by infecting the parental cells with lenti-Cas9 lentiviral particles, followed by selection of the infected cells with 10 ug/mL blasticidin (Thermo Fisher Scientific, R210-01). The

Cas9 stable A549 cells were then infected with packaged lentivirus pLenti-CRISPRv2 expressing small guide RNA (sgRNA) of interest, followed by treatment with 10 ug/mL puromycin (Bioshop, PUR333.500). The sequence of sgRNAs for targeting the coding region of the gene of interest are listed in SI Appendix 8.

Clonal cell lines were analyzed by WB for the absence of the protein and further analyzed for indel mutations within the targeted alleles by PCR. PCR products were cloned using the Zero Blunt PCR Cloning Kit (Thermo Fisher Scientific, K270040) and 10 clones were sequenced per cell line. The primers used for the PCR genotyping are listed in *SI Appendix,* Table*.* S1

Lentivirus production. Lentivirus pseudovirions were produced by transfecting HEK293T cells using Lipofectamine 2000 and 10 µg shRNAs, lenti-Cas9-Blast, pLenti-CRISPRv2 inserted with CRISPR guide RNA (gRNA) for gene knockout plasmids, or Lenti-ORF plasmid, 6 µg psPAX2 (Addgene, plasmid 12260) and 4 µg pMD2.G (Addgene, plasmid 12259). 48 h post-transfection, cell culture supernatant was collected from which pseudovirions were purified by ultracentrifugation (32,000 rpm) for 2 h. The pelleted virus was resuspended into DMEM medium. Virus titer was adjusted to 5 multiplicity of infection (MOI).

VSVΔ51-GFP virus infections. GFP-expressing VSVΔ51-GFP was previously described(58). Virus titer was determined using a standard plaque assay protocol(59). Virus replication was assessed *via* fluorescence microscopy. Viral MOIs used in each assay is described in the corresponding figure legends.

SARS-CoV-2 infection. SARS-CoV-2 (Genbank accession no. 599736; lineage B1.1.147) culture and related experiments were performed following Biological Safety Containment level 3 procedures. Virus stocks were generated and titrated via plaque assay using Vero E6 cells. The shCTR, sh4EHP#1-2, and shGIGYF2#1-2 Calu-3 cells were first challenged with poly(I:C) prior to infection. After 6 h of poly(I:C) stimulation, the cells were washed by PBS and EMEM containing 1% FBS was added to the cultures. Subsequently, the cells were infected with 0.01 or 0.05 MOI SARS-CoV-2. At 2 h post-infection, the cells were washed again and cultured in fresh EMEM containing 1% FBS. After 24 h culture, the supernatants were collected and treated with 10% triton X-100 for 30 min and used for ELISA assay. The cells were lysed by Trizol, followed by mRNA extraction and RT-qPCR to evaluate the expression of viral mRNAs.

Pattern Recognition Receptors (PRRs) ligands treatment and ELISA. Calu-3, HEK293 and A549 cells were seeded at 50-60% confluency. Cells were treated for 6 h with the appropriate concentration of either poly(I:C) (Sigma, P1530) or High Molecular Weight (HMW) poly(I:C) (InvivoGen, tlrl-pic) using Lipofectamine2000. IFN-β amounts in the culture supernatant were

measured by human IFN-β ELISA kit (R&D Systems, DIFNB0) according to the manufacturer's protocol.

RNA extraction and RT-PCR. Cells were harvested and RNA was isolated using mammalian total RNA isolation kit (Sigma, RTN70-1KT). Following the Superscript III reverse transcriptase protocol (Invitrogen), equal amounts of total RNA (1 µg) were used for reverse transcription with 100 ng random primers. mRNA abundance was estimated *via* real-time PCR system (Mastercycler Realplex, Eppendorf) using SYBR Green master mix (Bio-rad). All primers are listed in SI Appendix 8.

Plasmid construction. Synthesized viral coding sequences were incorporated into Gateway-compatible Entry vectors; pDONR207 SARS-CoV-2 NSP1 (Addgene, 141255), pDONR223 SARS-CoV-2 NSP2 (Addgene, 141256), pDONR207 SARS-CoV-2 E (Addgene, 141273) and expression clones with N-terminal fusion tags were produced simply by Gateway cloning (Gateway™ LR Clonase™ II Enzyme mix, Invitrogen, 11791020). The pcDNA3-FLAG-NSP1, FLAG-NSP2 and FLAG-E were constructed by using the pcDNA3-FLAG-gate-pGK-HYG (Addgene, 107397), as the destination vector. The Lenti-NSP2 was constructed by using the destination vector, pLenti-X2-Zeo-DEST (749-3) [a gift from Eric Campeau (Addgene, 21562)] and the donor vector, pDONR223 SARS-CoV-2 NSP2. The Lenti-NSP2 plasmid was used to generate NSP2 stable cell lines by following the lentivirus production method section. To generate the v5-tagged GIGYF2 fragment expression plasmids, the GIGYF2 fragments (A-F) were amplified by PCR from the previously described pcDNA3-FLAG-GIGYF2 plasmid(20) and sub-cloned into the PCI-neo-λN-v5 vector using XhoI and NotI restriction sites. The sequence of the primers used are listed in *SI Appendix,* Table. S1.

Dual Luciferase reporter assays. The *Ifnb1* promoter induced luciferase assay were descripted before(26). Briefly, the IFN-β–Luc was co-transfected with pRL-TK in wild type and 4EHP-KO HEK293 cells using Lipofectamine 2000 according to the manufacturer´s protocol (Invitrogen). 24 h after transfection, cells were lysed. Lysates were used to measure the activity of *F-Luc* and *R-Luc via* the Dual-Luciferase Reporter Assay System (Promega, E1960) in a GloMax 20/20 luminometer (Promega, USA) according to the manufacturer's instructions.

We adapted a previously used ISRE promoter-induced Luciferase assay in this study(26). The ISRE-Luc was co-transfected with pRL-TK in control, 4EHP-KO and GIGYF2-KO HEK293 cell lines. 16 h post-transfection, cells were treated with different concentrations of recombinant human IFN-β (R&D Systems, 8499-IF-010) for 12 h. Relative *F-Luc* activity compared to *R-Luc* was quantified using a dual luciferase assay (Promega, E1960).

The psiCHECK-2 control vector (Promega, C8021) and the psiCHECK-RL-*Ifnb1* 3´ UTR vector were described before(26). Briefly, the 3´ UTR sequence of human *Ifnb1* mRNA was inserted into the XhoI and NotI restriction sites in the *psiCHECK-2* vector downstream of the *Renilla luciferase* ORF(26). The encoded *R-Luc* mRNA contained a short 5´ UTR sequence (gctagccacc), which was similar in the control psiCHECK-2 and psiCHECK2-RL-*Ifnb1* 3´ UTR vectors. WT, 4EHP-KO or GIGYF2-KO HEK293 cells (150,000 cells/well) were co-transfected with either 10 ng *psiCHECK-2* reporter (as control) or a construct of luciferase with full length *Ifnb1* 3´ UTR (psiCHECK2-RL-*Ifnb1* 3´ UTR) using Lipofectamine 2000. 24 h post-transfection, cells were lysed, followed by dual-luciferase assay. *R-Luc* values were normalized against *F-Luc* levels for each sample.

For the tethering assay, we used the λN-BoxB tethering approach(39). Briefly, a *R-Luc* reporter containing five BoxB hairpins in its 3′ UTR was used. The 3′ end of the reporter contains a self-cleaving Hammerhead Ribozyme (HhR) to generate an internalized poly(a) stretch to prevent deadenylation and subsequent degradation(38). The reporter was co-transfected along with the construct encoding a fusion of the protein of interest ORF, like GIGYF2, to a λN peptide, which allows GIGYF2 to bind to reporter BoxB elements. HEK293 cells were co-transfected with the constructs expressing either λN-V5 control or λN-V5 fused with protein of interest along with *R-Luc-5boxB*-A114-N40-HhR or *R-Luc*-A114-N40-HhR, and *F-Luc*, followed by the dual-luc assay after 24 h transfection. The silencing effect of *R-Luc* (normalized by *F-Luc*) mediated by the protein of interest was examined by comparing the dual-luciferase activities of the cells.

Immunoprecipitation. Cells were washed with cold PBS and collected by scraping in the lysis buffer (40 mM HEPES pH 7.5, 120 mM NaCl, 1 mM EDTA, 50 mM NaF, 0.3% CHAPS, supplemented with complete EDTA-free protease inhibitor tablet and phosphatase inhibitor cocktail). 700 µg pre-cleared lysates were incubated with 1 µg anti-FLAG antibody and 50 µl Protein G agarose beads slurry per IP (Millipore) at 4 ℃ overnight. Beads were washed 3x10 mins with wash buffer (50 mM HEPES pH 7.5, 150 mM NaCl, 1 mM EDTA, 50 mM NaF, 0.3% CHAPS, supplemented with a complete EDTA-free protease inhibitor tablet and phosphatase inhibitor cocktail) and protein was eluted in SDS sample buffer.

Quantification and Statistical Analyses. Statistical tests were performed using Prism 6 (GraphPad). Error bars represent standard deviation (SD) from the mean. Number of independent replicates (≥3) and the statistical analysis used for each assay is described in the relevant figure legends. P values <0.05 were considered significant.

Figure. S1

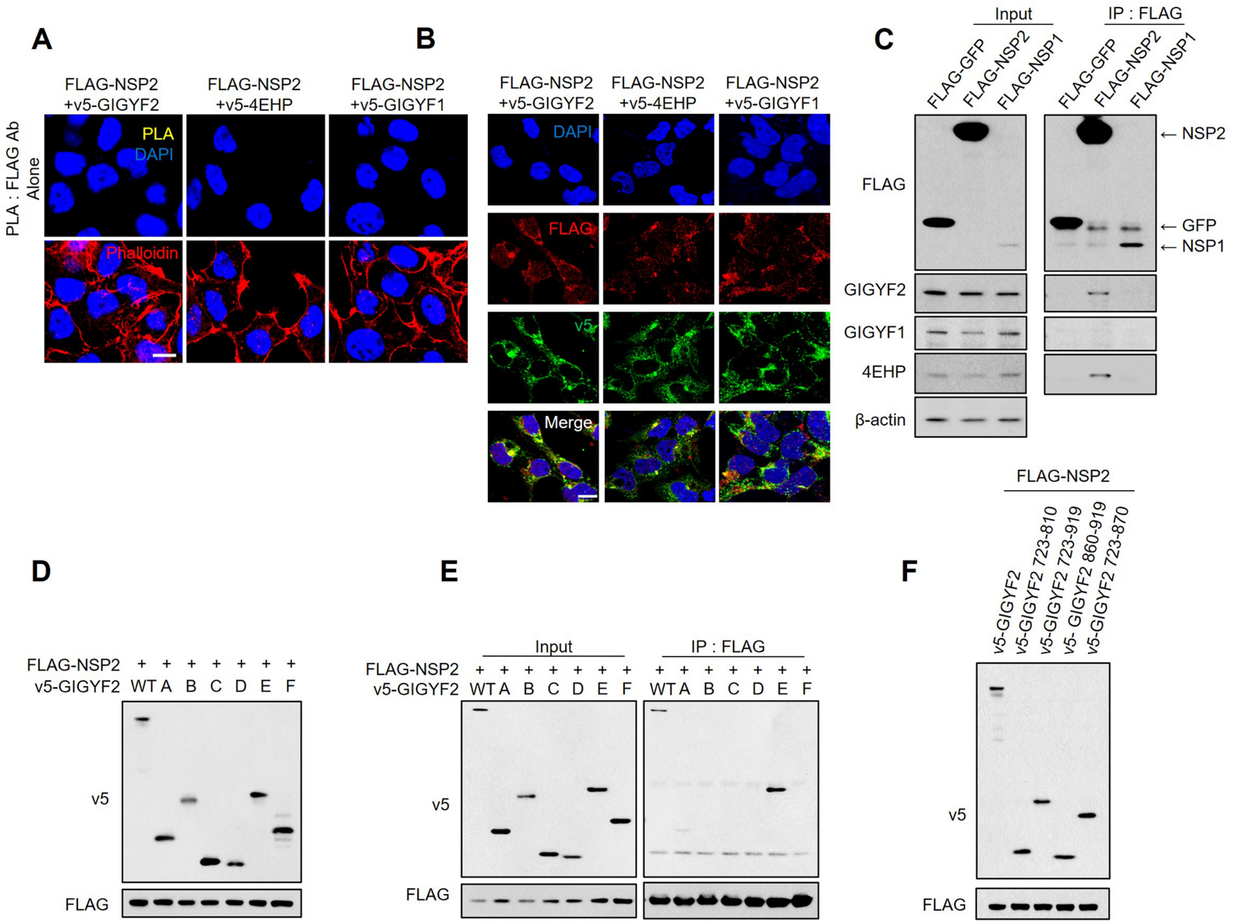
A
FLAG-NSP2 +v5-GIGYF2
FLAG-NSP2 +v5-4EHP
FLAG-NSP2 +v5-GIGYF1
PLA : FLAG Ab Alone
PLA
DAPI
Phalloidin
B
FLAG-NSP2 +v5-GIGYF2
FLAG-NSP2 +v5-4EHP
FLAG-NSP2 +v5-GIGYF1
DAPI
FLAG
v5
Merge
C
Input
IP : FLAG
FLAG-GFP
FLAG-NSP2
FLAG-NSP1
FLAG-GFP
FLAG-NSP2
FLAG-NSP1
FLAG
GIGYF2
GIGYF1
4EHP
β-actin
← NSP2
← GFP
← NSP1
FLAG-NSP2
v5-GIGYF2
v5-GIGYF2 723-810
v5-GIGYF2 723-919
v5- GIGYF2 860-919
v5-GIGYF2 723-870
D
FLAG-NSP2 + + + + + + +
v5-GIGYF2 WT A B C D E F
v5
FLAG
E
Input
IP : FLAG
FLAG-NSP2 + + + + + + + + + + + + + +
v5-GIGYF2 WT A B C D E F WT A B C D E F
v5
FLAG
F
v5
FLAG

Figure S1. Immunofluorescence visualisation of markers used in PLA experiments and Co-IP experiments; related to Figure 1. (*A*) Negative controls represent PLA performed with a single antibody (PLA: FLAG ab alone), related to Fig. 1*A*. Scale bar= 10 μm, n=5 independent replicates. (*B*) Immunofluorescence staining with the indicated antibodies in cells shown in Fig. 1*A*. Scale bar= 10 μm. (*C*) HEK293T cells were transfected with vectors expressing FLAG-tagged SARS-CoV-2 NSP2, NSP1, or GFP. Proteins were immunoprecipitated with an anti-FLAG antibody, followed by western blot with the indicated antibodies. (*D*) Expression of the full-length GIGYF2 and its fragments shown in Fig. 1*D* was measured by western blotting. (*E*) Co-IP assay for detection of interaction between Flag-NSP2 and full-length (FL) or truncated isoforms of v5-GIGYF2 in HEK293T cells. Whole-cell lysates were prepared 24 h after transfection and subjected to immunoprecipitation using anti-FLAG antibody followed by blotting with the indicated antibodies. (*F*) Expression of indicated GIGYF2-LHR fragments shown in Fig. 1*F*. was measured by western blotting. The number of PLA signals from at least 20 cells was counted in each sample. Data are presented as mean ± SD (n=5).

Figure. S2

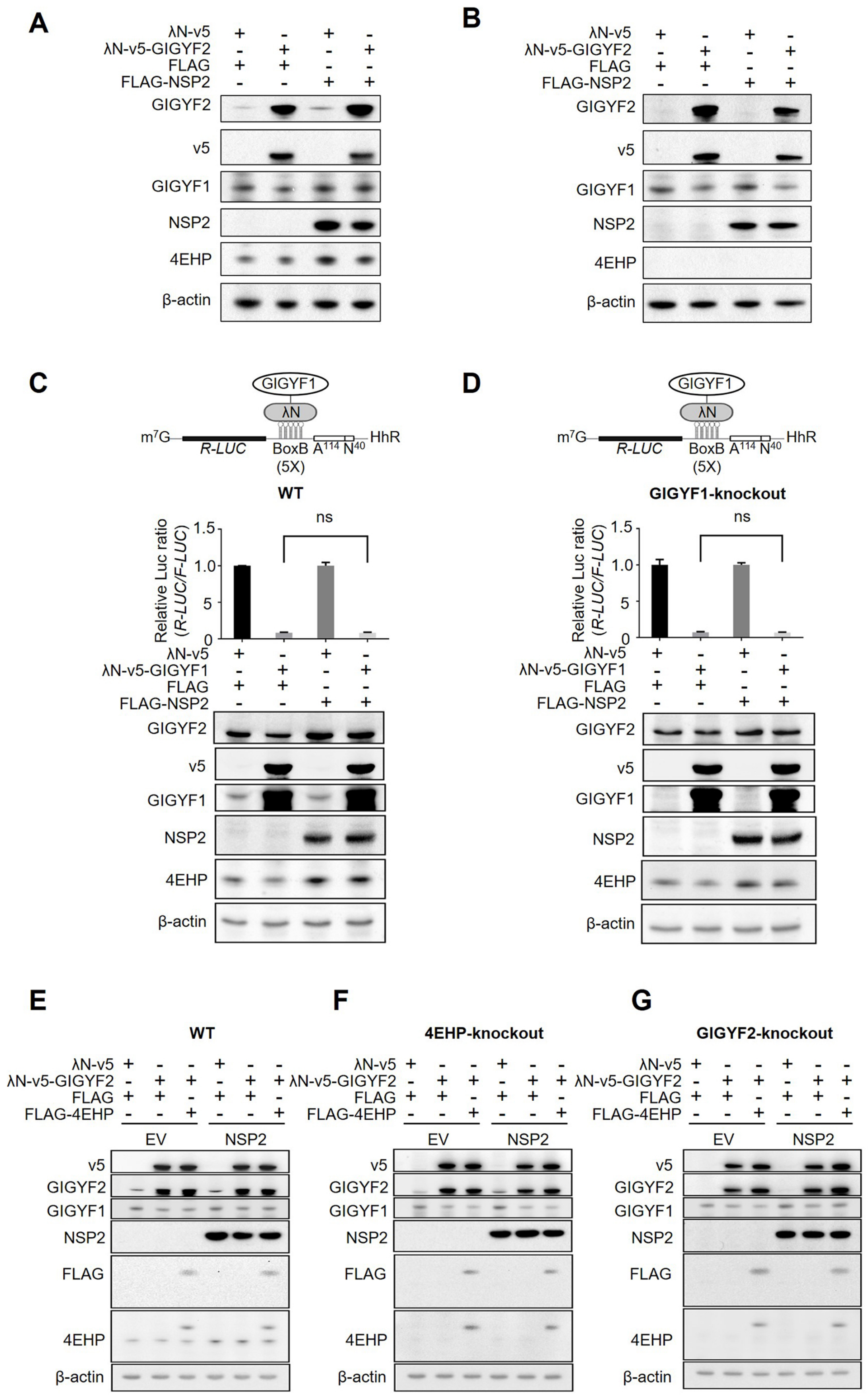

A
λN-v5
λN-v5-GIGYF2
FLAG
FLAG-NSP2
GIGYF2
v5
GIGYF1
NSP2
4EHP
β-actin
B
λN-v5
λN-v5-GIGYF2
FLAG
FLAG-NSP2
GIGYF2
v5
GIGYF1
NSP2
4EHP
β-actin
C
GIGYF1
λN
m7G
R-LUC
BoxB (5X)
A114 N40
HhR
WT
ns
Relative Luc ratio (R-LUC/F-LUC)
1.5
1.0
5
0
λN-v5
λN-v5-GIGYF1
FLAG
FLAG-NSP2
GIGYF2
v5
GIGYF1
NSP2
4EHP
β-actin
D
GIGYF1
λN
m7G
R-LUC
BoxB (5X)
A114 N40
HhR
GIGYF1-knockout
ns
Relative Luc ratio (R-LUC/F-LUC)
1.5
1.0
5
0
λN-v5
λN-v5-GIGYF1
FLAG
FLAG-NSP2
GIGYF2
v5
GIGYF1
NSP2
4EHP
β-actin
E
WT
λN-v5
λN-v5-GIGYF2
FLAG
FLAG-4EHP
EV
NSP2
v5
GIGYF2
GIGYF1
NSP2
FLAG
4EHP
β-actin
F
4EHP-knockout
λN-v5
λN-v5-GIGYF2
FLAG
FLAG-4EHP
EV
NSP2
v5
GIGYF2
GIGYF1
NSP2
FLAG
4EHP
β-actin
G
GIGYF2-knockout
λN-v5
λN-v5-GIGYF2
FLAG
FLAG-4EHP
EV
NSP2
v5
GIGYF2
GIGYF1
NSP2
FLAG
4EHP
β-actin

Figure S2. Western blot analysis of the GIGYF1/2 tethering experiments; related to Figure 2. (*A* **&** *B*) Western blot with the indicated antibodies using lysates from the cell shown in Fig. 2*A* and Fig. 2*B*, respectively. (*C*) GIGYF1 tethering dual-luciferase assay in the presence or absence of FLAG-NSP2 was performed in WT HEK293 cells (upper panel) and cell lysate was used for western blotting (lower panel). Data are presented as mean ± SD (n=3). ns= non-significant, one-way ANOVA with Bonferroni's post hoc test. (*D*) GIGYF1 tethering dual-luciferase assay (upper panel) and western blot (lower panel) analysis were performed in GIGYF1-KO cells in the presence or absence of FLAG-NSP2. Data are presented as mean ± SD (n=3). (*E*-*G*) Western blotting with the indicated antibodies using lysates from the cells shown in Fig. 2*C*-*E*. ns= non-significant, one-way ANOVA with Bonferroni's post hoc test.

Figure. S3

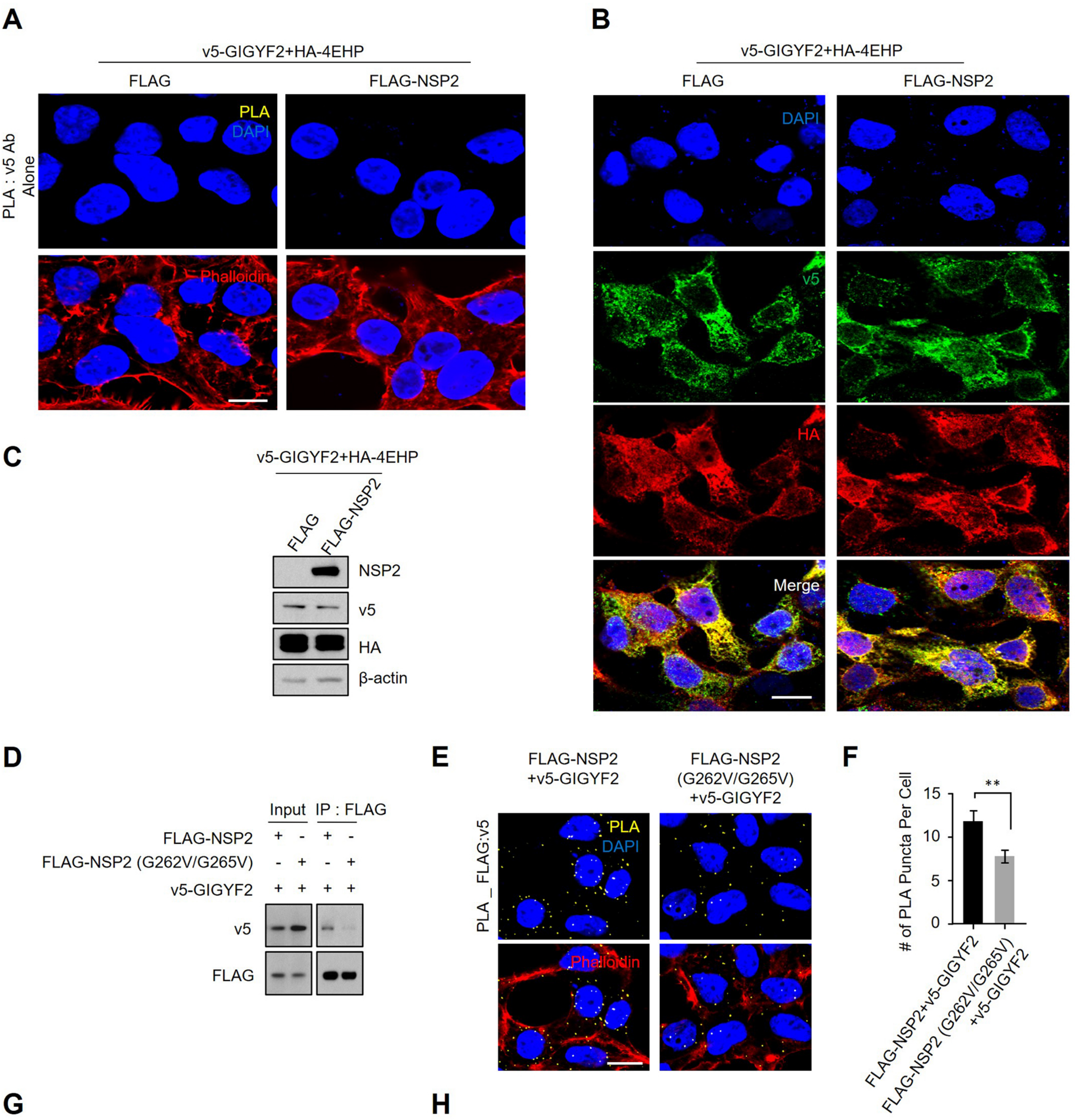
A
v5-GIGYF2+HA-4EHP
FLAG
FLAG-NSP2
PLA : v5 Ab Alone
PLA
DAPI
Phalloidin
B
v5-GIGYF2+HA-4EHP
FLAG
FLAG-NSP2
DAPI
v5
HA
Merge
C
v5-GIGYF2+HA-4EHP
FLAG
FLAG-NSP2
NSP2
v5
HA
β-actin
D
Input
IP : FLAG
FLAG-NSP2
FLAG-NSP2 (G262V/G265V)
v5-GIGYF2
v5
FLAG
E
FLAG-NSP2 +v5-GIGYF2
FLAG-NSP2 (G262V/G265V) +v5-GIGYF2
PLA _ FLAG:v5
PLA
DAPI
Phalloidin
F
# of PLA Puncta Per Cell
15
10
5
0
**
FLAG-NSP2+v5-GIGYF2
FLAG-NSP2 (G262V/G265V) +v5-GIGYF2
G
FLAG-NSP2
FLAG-NSP2 (G262V/G265V)
v5-GIGYF2
v5
FLAG
H
EV
NSP2 (WT)
NSP2 (MUT)
λN-v5
λN-v5-GIGYF2
GIGYF2
v5
NSP2
4EHP
β-actin

Figure S3. Immunofluorescence visualisation of markers used in PLA experiments and Co-IP experiments; related to Figure 2. (*A*) Negative controls represent PLA punctate performed with a single antibody (PLA: v5 ab alone). Scale bar= 10 μm, n=5 independent experiments. (*B*) Immunofluorescence staining performed with cells shown in Fig. 2*G*. Scale bar= 10 μm. (*C*) Western blot analysis of cells shown in Fig. 2*G*. **(***D***)** Co-IP assay with v5-GIGYF2 and FLAG-NSP2 or FLAG-NSP2$^{G262V/G265V}$ using anti-FLAG antibody in HEK293T cells. **(***E***)** PLA assay for investigating the interaction between v5-GIGYF2and FLAG-NPS2 or FLAG- FLAG-NSP2$^{G262V/G265V}$ as described in (d). **(***F***)** Quantification of positive PLA signals from (*E*). (*G*) Western blot analysis of cells shown in (e). (*H*) Western blot analysis of cells shown in Fig. 2*I*. The number of PLA signals from at least 20 cells was counted in each sample. Data are presented as mean ± SD (n=5). **P< 0.01, one-way ANOVA with Bonferroni's post hoc test.

Figure. S4

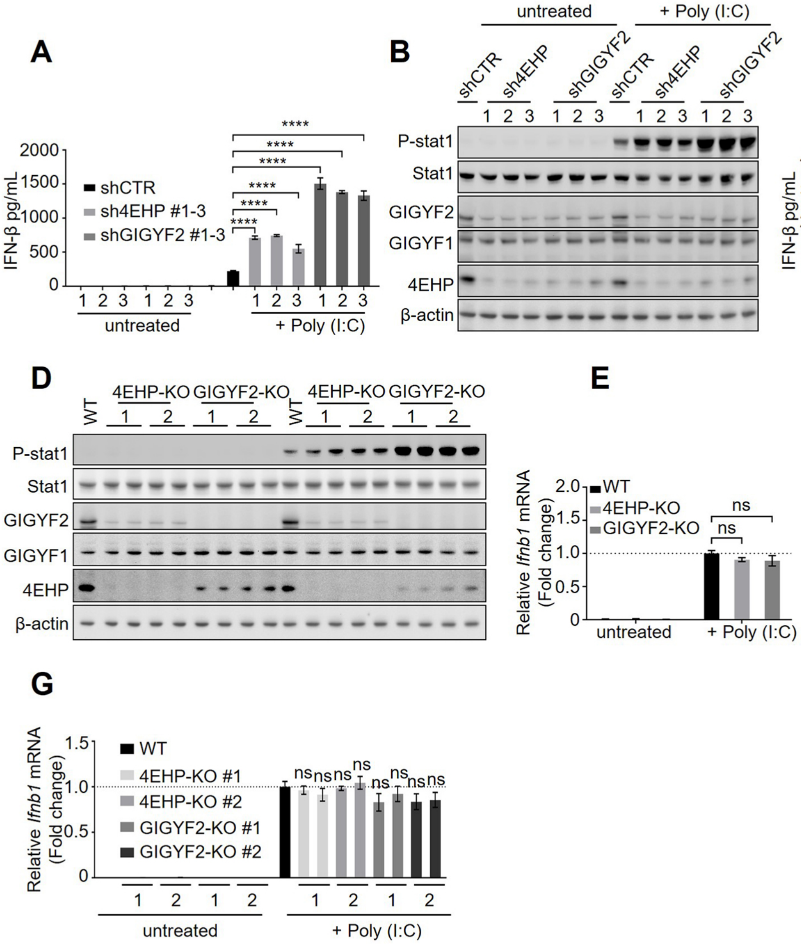

A
IFN-β pg/mL
shCTR
sh4EHP #1-3
shGIGYF2 #1-3
untreated
+ Poly (I:C)
B
untreated
+ Poly (I:C)
shCTR
sh4EHP
shGIGYF2
P-stat1
Stat1
GIGYF2
GIGYF1
4EHP
β-actin
C
IFN-β pg/mL
WT
4EHP-KO #1
4EHP-KO #2
GIGYF2-KO #1
GIGYF2-KO #2
untreated
+ Poly (I:C)
D
WT
4EHP-KO
GIGYF2-KO
P-stat1
Stat1
GIGYF2
GIGYF1
4EHP
β-actin
E
Relative Ifnb1 mRNA (Fold change)
WT
4EHP-KO
GIGYF2-KO
ns
untreated
+ Poly (I:C)
F
Relative Ifnb1 mRNA (Fold change)
shCTR
sh4EHP #1-3
shGIGYF2 #1-3
ns
untreated
+ Poly (I:C)
G
Relative Ifnb1 mRNA (Fold change)
WT
4EHP-KO #1
4EHP-KO #2
GIGYF2-KO #1
GIGYF2-KO #2
ns
untreated
+ Poly (I:C)

Figure S4. GIGYF2/4EHP complex represses IFN-β production without affecting *Ifnb1* mRNA level; related to Figure 3. (*A*) ELISA measurement of IFN-β production in shCTR, sh4EHP#1-3 and shGIGYF2#1-3 in Calu-3 cells 6 h post-treatment with 0.5 μg/mL poly(I:C). Data are presented as mean ± SD (n=3). ****P< 0.0001, one-way ANOVA with Bonferroni's post hoc test. **(*B*)** Western blot analysis of cell lysates from (*A*). **(*C*)** ELISA measurements of IFN-β production in WT, 4EHP-KO#1-2, and GIGYF2-KO#1-2 A549 cells after 6 h treatment with 1 μg/mL poly(I:C). Data are presented as mean ± SD (n=3). ***P< 0.001, ****P< 0.0001, one-way ANOVA with Bonferroni's post hoc test. **(*D*)** Western blot analysis of cell lysates from (*C*). **(*E*)** RT-qPCR analysis of WT, 4EHP-KO, and GIGYF2-KO HEK293 cell lines transiently expressing TLR3 (treatment described in Fig. 3*A*) 6 h post-treatment with poly(I:C). **(*F*)** RT-qPCR analysis of shCTR, sh4EHP#1-3, and shGIGYF2#1-3 Calu-3 cells treated with poly(I:C). **(*G*)** RT-qPCR analysis of WT, 4EHP-KO#1-2, and GIGYF2-KO#1-2 A549 cell lines 6 h post-treatment with poly(I:C).

Figure. S5

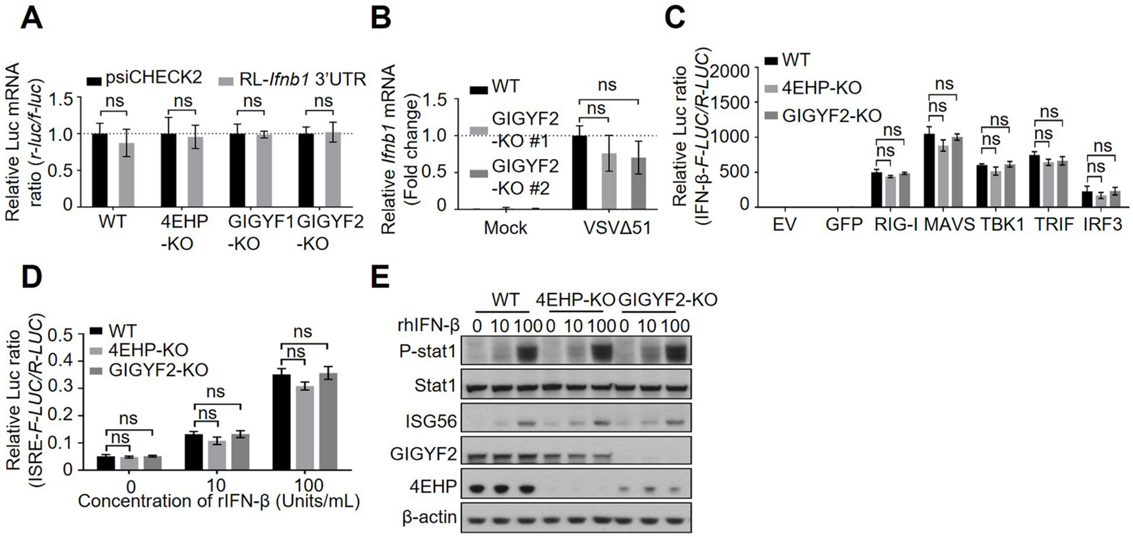
A
psiCHECK2
RL-Ifnb1 3'UTR
Relative Luc mRNA ratio (r-luc/f-luc)
1.5
1.0
0.5
0
ns
WT
4EHP -KO
GIGYF1 -KO
GIGYF2 -KO
B
WT
GIGYF2 -KO #1
GIGYF2 -KO #2
Relative Ifnb1 mRNA (Fold change)
Mock
VSVΔ51
C
WT
4EHP-KO
GIGYF2-KO
Relative Luc ratio (IFN-β-F-LUC/R-LUC)
2000
1500
1000
500
EV
GFP
RIG-I
MAVS
TBK1
TRIF
IRF3
D
WT
4EHP-KO
GIGYF2-KO
Relative Luc ratio (ISRE-F-LUC/R-LUC)
0.5
0.4
0.3
0.2
0.1
0
10
100
Concentration of rIFN-β (Units/mL)
E
WT
4EHP-KO
GIGYF2-KO
rhIFN-β 0 10 100 0 10 100 0 10 100
P-stat1
Stat1
ISG56
GIGYF2
4EHP
β-actin

Figure S5. GIGYF2/4EHP complex does not directly affect the signalling pathways upstream and downstream of IFN-β; related to Figure 3. (*A*) RT-qPCR measurement of the *R-Luc* mRNA levels described in Fig. 3*G*. The *Luciferase* mRNA levels in the empty vector were used for normalization. Data are presented as mean ± SD (n=3). ns=non-significant, two-way ANOVA with Bonferroni's post hoc test. **(***B***)** RT-qPCR analysis of *Ifnb1* mRNA in the WT or GIGYF2-KO#1-2 A549 cells following VSVΔ51-GFP virus infection, as described in Fig. 3*F*. **(***C***)** WT, 4EHP-KO, or GIGYF2-KO HEK293 cells were co-transfected with *Ifnb1* promoter-driven *F-Luc* (*IFN-β-Luc*) and *R-Luc* expression plasmids 24 h post-transfection with empty vector, GFP, RIG-I, MAVS, TBK1, TRIF, or IRF3. Luciferase activities were measured 16 h after the 2nd transfection. **(***D***)** The WT, 4EHP-KO, and GIGYF2-KO HEK293 cells were co-transfected with both *ISRE-F-Luc* and *R-Luc* plasmids. 16 h post-transfection, cells were treated with indicated concentrations of recombinant human IFN-β for 12 h. The *ISRE-F-Luc*/*R-Luc* ratios were quantified by dual luciferase assay. ns=non-significant. **(***E***)** Western blot analysis of cell lysates from (*D*).

Figure. S6

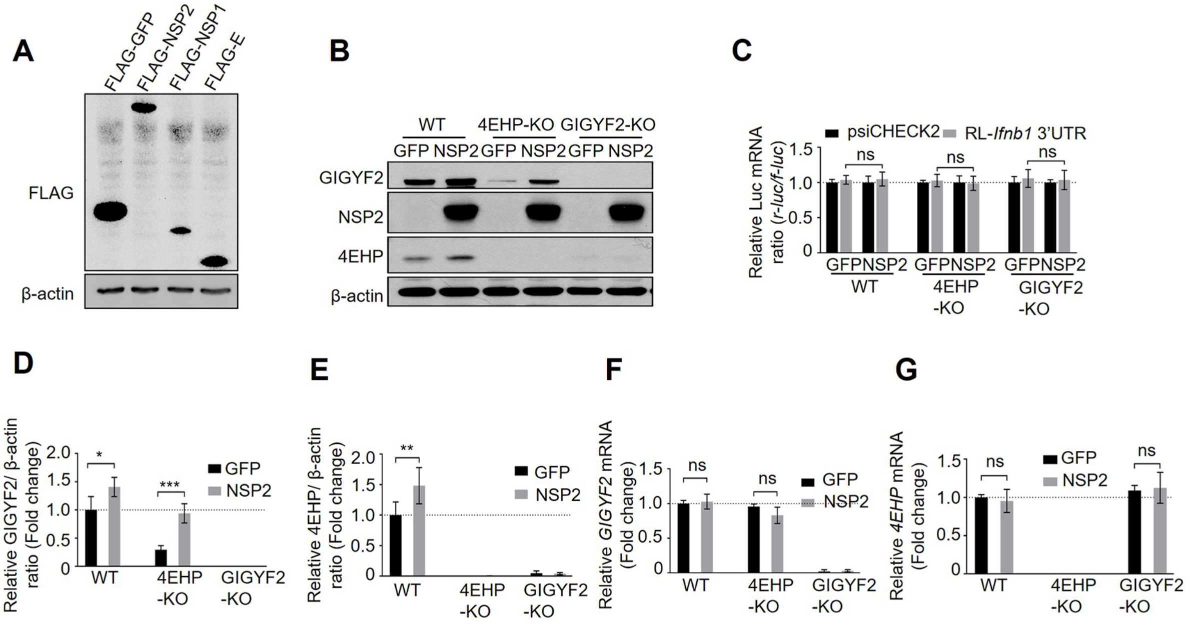
A
FLAG-GFP
FLAG-NSP2
FLAG-NSP1
FLAG-E
FLAG
β-actin
B
WT
4EHP-KO
GIGYF2-KO
GFP NSP2 GFP NSP2 GFP NSP2
GIGYF2
NSP2
4EHP
β-actin
C
psiCHECK2
RL-*Ifnb1* 3'UTR
Relative Luc mRNA ratio (*r-luc/f-luc*)
ns
GFP NSP2
WT
4EHP -KO
GIGYF2 -KO
D
Relative GIGYF2/ β-actin ratio (Fold change)
GFP
NSP2
*
***
WT
4EHP -KO
GIGYF2 -KO
E
Relative 4EHP/ β-actin ratio (Fold change)
**
GFP
NSP2
F
Relative *GIGYF2* mRNA (Fold change)
ns
G
Relative *4EHP* mRNA (Fold change)

Figure S6. Ectopic expression of NSP2 enhances endogenous GIGYF2 and 4EHP protein expression without altering the *GIGYF2* and *4EHP* mRNA abundance; Related to Figure 4. **(*A*)** Western blot analysis of cell lysates from Fig. 4*A*. (*B*) Western blot analysis of the indicated proteins in cell lysates from Fig. 4*B*. **(*C*)** RT-qPCR measurement of mRNA levels of the luciferase reporters described in Fig. 4*B*. The luciferase mRNA levels in empty vector were used for normalization. **(*D*-*E*)** Quantitation of expression of endogenous GIGYF2 (*D*) or 4EHP (*E*). β-actin expression was used as internal control. **(*F*-*G*)** RT-qPCR analysis of *GIGYF2* mRNA (*F*) or *4EHP* mRNA (*G*) levels. The WT HEK293 cells overexpressing GFP was used for normalization. Data are presented as mean ± SD (n=3). ns=non-significant, two-way ANOVA with Bonferroni's post hoc test.

Figure. S7

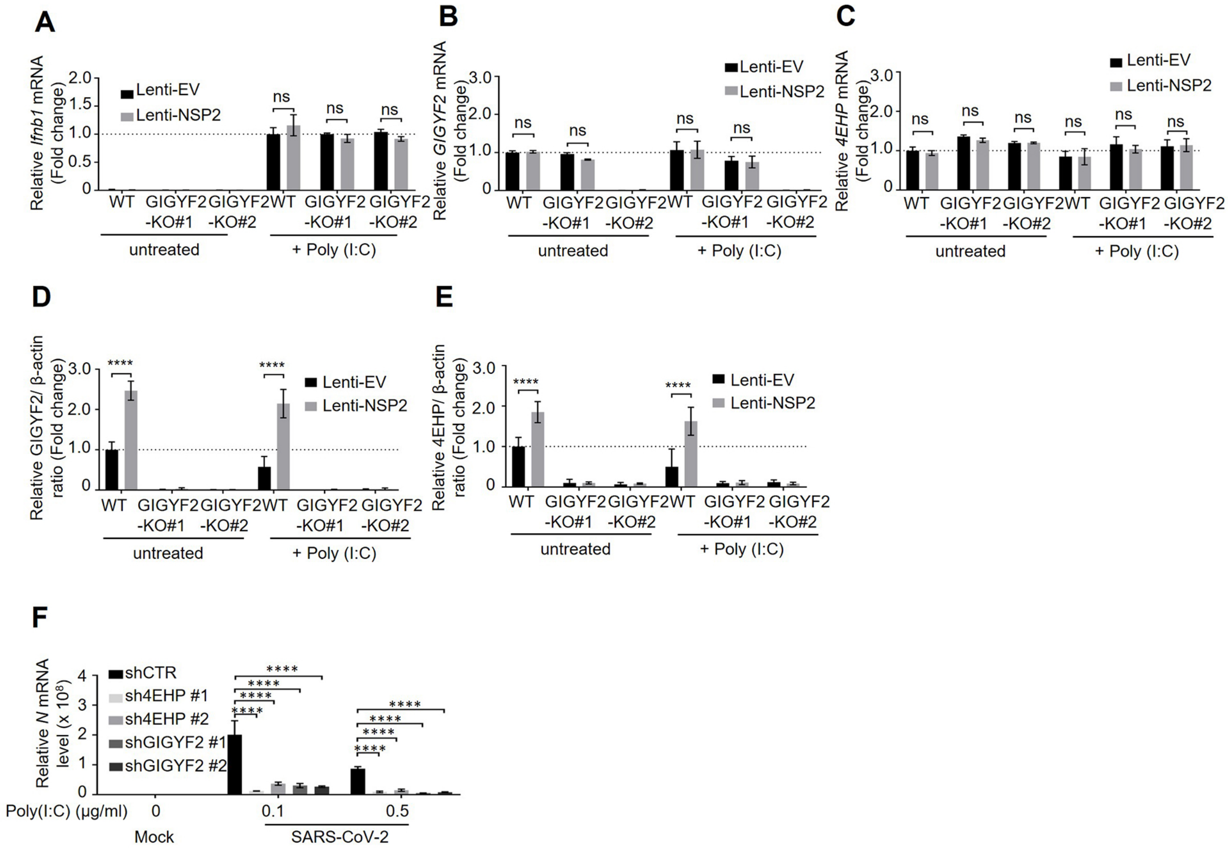
A
Relative Ifnb1 mRNA (Fold change)
Lenti-EV
Lenti-NSP2
ns
WT
GIGYF2 -KO#1
GIGYF2 -KO#2
untreated
+ Poly (I:C)
B
Relative GIGYF2 mRNA (Fold change)
C
Relative 4EHP mRNA (Fold change)
D
Relative GIGYF2/ β-actin ratio (Fold change)
****
E
Relative 4EHP/ β-actin ratio (Fold change)
F
Relative N mRNA level (x 10^8)
shCTR
sh4EHP #1
sh4EHP #2
shGIGYF2 #1
shGIGYF2 #2
Poly(I:C) (µg/ml)
0
0.1
0.5
Mock
SARS-CoV-2

Figure S7. NSP2/GIGYF2/4EHP repressor complex does not affect *Ifnb1* mRNA abundance, Related to Figure 4. (*A*-*C*) RT-qPCR analysis of *Ifnb1* (*A*), *GIGYF2* (*B*), *and 4EHP* (*C*) mRNAs from samples described in Fig. 4*C*. **(*D*-*E*)** Quantitation of expression of endogenous GIGYF2 (*D*) and 4EHP (*E*) proteins from samples described in Fig. 4*D*. (*F*) Relative viral *N* mRNA expression level from the described samples in Fig. 4*E* was measured by RT-qPCR, normalized to the *GAPDH* mRNA, and expressed as fold values relative to the Mock-infected shCTR cells. Data are presented as mean ± SD (n=3). ns=non-significant, ****P< 0.0001, two-way ANOVA with Bonferroni's post hoc test.

Table S1

Name	Sequence	Application
IFN-β-Fwd	5'-AAACTCATGAGCAGTCTGCA-3'	RT-PCR
IFN-β-Rv	5'-AGGAGATCTTCAGTTTCGGAGG-3'	RT-PCR
GAPDH-Fwd	5'-TGGGTGTGAACCATGAGAAG-3'	RT-PCR
GAPDH-Rv	5'-ATGGACTGTGGTCATGAGTC-3'	RT-PCR
ISG56-Fwd	5'-TCCCCTAAGGCAGGCTGTC-3'	RT-PCR
ISG56-Rv	5'-GACATGTTGGCTAGAGCTTCTTC-3'	RT-PCR
eIF4E2-Fwd	5'-TCCCGTTGCTGGGAGAATCTCA-3'	RT-PCR
eIF4E2-Rv	5'-GTTGCTTGGTCACTGGCAGTCT-3'	RT-PCR
GIGYF1-Fwd	5'-GCTGCCGTGGAGATTCGAGAAAGC-3'	RT-PCR
GIGYF1-Rv	5'-CCCGCAGCCTCAGCTCCTGGC-3'	RT-PCR
GIGYF2-Fwd	5'-GGGGGACATAGCCGTGATGCCTTTTAAG-3'	RT-PCR
GIGYF2-Rv	5'-GGTGGAAAGGCATGGGTCAATACATTAG-3'	RT-PCR
GIGYF2-Fwd	5'-GAGAAGCTGGGGAGTATTGACTGGGG-3'	RT-PCR
GIGYF2-Rv	5'-CCCCTCCCCAAGAATCACTCTTCC-3'	RT-PCR
GIGYF2-Fwd	5'-AACGACTGACCAGGCAGCAAGA-3'	RT-PCR
GIGYF2-Rv	5'-GGAAGACAGTGCTGCTTTCTGC-3'	RT-PCR
Firefly luciferase-Fwd	5'-GCCATGAAGCGCTACGCCCTGG-3'	RT-PCR
Firefly luciferase-Rv	5'-TCTTGCTCACGAATACGACGGTGG-3'	RT-PCR
Renilla luciferase-Fwd	5'-TCAGTGGTGGGCTCGCTGCA-3'	RT-PCR
Renilla luciferase-Rv	5'-CTTTGGAAGGTTCAGCAGCTCG-3'	RT-PCR
CoV-2 S-Fwd1	5'-CAATGGTTTAACAGGCACAGG-3'	RT-PCR
CoV-2 S-Rv1	5'-CTCAAGTGTCTGTGGATCACG-3'	RT-PCR
CoV-2 S-Fwd2	5'-TCCTGGTGATTCTTCTTCAGGT-3'	RT-PCR
CoV-2 S-Rv2	5'-TCTGAGAGAGGGTCAAGTGC-3'	RT-PCR
CoV-2 N-Fwd	5'-GACCCCAAAATCAGCGAAAT-3'	RT-PCR
CoV-2 N-Rv	5'-TCTGGTTACTGCCAGTTGAATCTG-3'	RT-PCR
GIGYF1 sgRNA#1-Fwd	5'-CACCGTGACTACCGTTATGGGCGAG-3'	Cloning
GIGYF1 sgRNA#1-Rv	5'-AAACCTCGCCCATAACGGTAGTCAC-3'	Cloning
GIGYF1 sgRNA#2-Fwd	5'-CACC AGCTGGCTGACTACCGTTAT-3'	Cloning
GIGYF1 sgRNA#2-Rv	5'-AAACATAACGGTAGTCAGCCAGCTC-3'	Cloning
GIGYF1 sgRNA#3-Fwd	5'-CACCGAAGCTGGCTGACTACCGTTA-3'	Cloning
GIGYF1 sgRNA#3-Rv	5'-AAACTAACGGTAGTCAGCCAGCTTC-3'	Cloning
GIGYF2 sgRNA#1-Fwd	5'-CACCGGGATGTAATACTCCCACCAC-3'	Cloning
GIGYF2 sgRNA#1-Rv	5'-AAACGTGGTGGGAGTATTACATCCC-3'	Cloning
GIGYF2 sgRNA#2-Fwd	5'-CACCGAATTTATACTTCGGCAATGC-3'	Cloning
GIGYF2 sgRNA#2-Rv	5'-AAACGCATTGCCGAAGTATAAATTC-3'	Cloning
GIGYF2 sgRNA#3-Fwd	5'-CACCGGGAGGAACCCCTTCCACCAT-3'	Cloning
GIGYF2 sgRNA#3-Rv	5'-AAACATGGTGGAAGGGGTTCCTCCC-3'	Cloning
GIGYF2-A-XHO1-Fwd	5'-GAGCTCGAGATGGCAGCGGAAACGCAGAC-3'	Cloning
GIGYF2-A-NOT1-Rv	5'-GAGGCGGCCGCCTACCAGCCTGCAGAACGAGGGC-3'	Cloning
GIGYF2-B-XHO1-Fwd	5'-GAGCTCGAGAGTCCTGATGGCCCTCGTTC-3'	Cloning
GIGYF2-B-NOT1-Rv	5'-GAGGCGGCCGCCTAACCAGGAGCACCTACAACTG-3'	Cloning
GIGYF2-C-XHO1-Fwd	5'-GAGCTCGAGGTTGAAACACCAGTTGTAGG-3'	Cloning

GIGYF2-C-NOT1-Rv	5'-GAGGCGGCCGCCTACAGTCGTTCCTGGTCCAGCTC-3'	Cloning
GIGYF2-D-XHO1-Fwd	5'-GAGCTCGAGCATATGGGAGAGCTGGACCAG-3'	Cloning
GIGYF2-D-NOT1-Rv	5'-GAGGCGGCCGCCTACTTCTCTAGCTGCTGAAGCTG-3'	Cloning
GIGYF2-E-XHO1-Fwd	5'-GAGCTCGAGGCCCTGGAACAGCTTCAGCAG-3'	Cloning
GIGYF2-E-NOT1-Rv	5'-GAGGCGGCCGCCTAACTACTGACTAGGTCAGATGC-3'	Cloning
GIGYF2-F-XHO1-Fwd	5'-GAGCTCGAGAACCAGTGGGCATCTGACCT-3'	Cloning
GIGYF2-F-NOT1-Rv	5'-GAGGCGGCCGCTCAGTAGTCATCCAACGTCTC-3'	Cloning
4EHP sgRNA#1 a	5'-TGAGCTCGTGGGACGGCCGG-3'	CRISPR
4EHP sgRNA#1 b	5'-TGAAGAGATGGAAGTCACTG-3'	CRISPR
4EHP sgRNA#1 c	5'-TTGTATTCCATAATGGTGTT-3'	CRISPR
4EHP sgRNA#2 a	5'-AAAAGTGTAGTTGTACTGCA-3'	CRISPR
4EHP sgRNA#2 b	5'-TTGTATTCCATAATGGTGTT-3'	CRISPR
4EHP sgRNA#2 c	5'-GGATATATTAATGGTTTCTTTTGG-3'	CRISPR
GIGYF2 sgRNA#1 a	5'-GGCAATGCTGGAGAAAGAGG-3'	CRISPR
GIGYF2 sgRNA#1 b	5'-AATACGGAAAAGAATGGCAG-3'	CRISPR
GIGYF2 sgRNA#1 c	5'-TGTCTTGGCCTGGTCGGAGGCATC-3'	CRISPR
GIGYF2 sgRNA#2 a	5'-GATTGGTCTGAAATACTGAA-3'	CRISPR
GIGYF2 sgRNA#2 b	5'-TGATGAACGGGGTTACCGAA-3'	CRISPR
GIGYF2 sgRNA#2 c	5'-CGCAGCTTAAGAAAAGTAGCCGTC-3'	CRISPR

Table S1. The list of primers and CRISPR guide RNA used in this study; related to methods section.
